# Comprehensive phylogenetic analysis of all species of swordtails and platies (Pisces: Genus *Xiphophorus*) uncovers a hybrid origin of a swordtail fish, *Xiphophorus monticolus*, and demonstrates that the sexually selected sword originated in the ancestral lineage of the genus, but was lost again secondarily

**DOI:** 10.1186/1471-2148-13-25

**Published:** 2013-01-29

**Authors:** Ji Hyoun Kang, Manfred Schartl, Ronald B Walter, Axel Meyer

**Affiliations:** 1 Lehrstuhl für Zoologie und Evolutionsbiologie, Department of Biology, University of Konstanz, Universitätsstraße 10, Konstanz 78457, Germany; 2 Konstanz Research School Chemical Biology, University of Konstanz, Konstanz, Germany; 3 Physiological Chemistry, Biozentrum, University of Würzburg, Am Hubland, Würzburg, 97074, Germany; 4 Department of Chemistry and Biochemistry, Texas State University-San Marcos, 601 University Dr, San Marcos, TX, 78666, USA

## Abstract

**Background:**

Males in some species of the genus *Xiphophorus*, small freshwater fishes from Meso-America, have an extended caudal fin, or sword – hence their common name “swordtails”. Longer swords are preferred by females from both sworded and – surprisingly also, non-sworded (platyfish) species that belong to the same genus. Swordtails have been studied widely as models in research on sexual selection. Specifically, the pre-existing bias hypothesis was interpreted to best explain the observed bias of females in presumed ancestral lineages of swordless species that show a preference for assumed derived males with swords over their conspecific swordless males. However, many of the phylogenetic relationships within this genus still remained unresolved. Here we construct a comprehensive molecular phylogeny of all 26 known *Xiphophorus* species, including the four recently described species (*X. kallmani*, *X. mayae*, *X. mixei* and *X. monticolus*). We use two mitochondrial and six new nuclear markers in an effort to increase the understanding of the evolutionary relationships among the species in this genus. Based on the phylogeny, the evolutionary history and character state evolution of the sword was reconstructed and found to have originated in the common ancestral lineage of the genus *Xiphophorus* and that it was lost again secondarily.

**Results:**

We estimated the evolutionary relationships among all known species of the genus *Xiphophorus* based on the largest set of DNA markers so far. The phylogeny indicates that one of the newly described swordtail species, *Xiphophorus monticolus*, is likely to have arisen through hybridization since it is placed with the southern platyfish in the mitochondrial phylogeny, but with the southern swordtails in the nuclear phylogeny. Such discordance between these two types of markers is a strong indication for a hybrid origin. Additionally, by using a maximum likelihood approach the possession of the sexually selected sword trait is shown to be the most likely ancestral state for the genus *Xiphophorus*. Further, we provide a well supported estimation of the phylogenetic relationships between the previously unresolved northern swordtail groups.

**Conclusions:**

This comprehensive molecular phylogeny of the entire genus *Xiphophorus* provides evidence that a second swordtail species, *X. monticolus*, arose through hybridization. Previously, we demonstrated that *X. clemenciae*, another southern swordtail species, arose via hybridization. These findings highlight the potential key role of hybridization in the evolution of this genus and suggest the need for further investigations into how hybridization contributes to speciation more generally.

## Background

Species in the genus *Xiphophorus* (Family Poeciliidae) are small live-bearing freshwater fish that are distributed from northern Mexico to Belize and Honduras [1,2]. Poeciliids have been widely studied in fields ranging from ecology, evolution, genetics, and genomics to systematics [3]. These fish have been investigated in an effort to improve our understanding of the evolution of several life-history and behavioral traits including viviparity [4,5], the placenta [6] and female mating preference for exaggerated male traits such as the sword. The sexually selected sword trait is generally assumed to have arisen through “sensory exploitation” and a “pre-existing bias” [7,8]. More recently, fish of this group have also been the foci of studies aimed at uncovering the genetic mechanisms underlying evolutionary processing during speciation [9-15].

The genus *Xiphophorus* is particularly interesting from an evolutionary perspective because several of its species have a unique morphological feature, the “sword”. The “sword-bearing” species of *Xiphophorus*, are called swordtails, and the others, that lack the sword, are called platyfish. Their males lack this male specific trait - a conspicuously colored elongation of the ventral rays of the caudal fin [7,16]. The investigation of this unique feature has provided many interesting evolutionary insights, particularly in regards to open issues in the field of sexual selection. The evolution of this exaggerated male trait appears to be detrimental to the males’ survival, since, although it is attractive to females, it also makes them more conspicuous to predators [17].

The pre-existing bias hypothesis [18,19] was proposed to explain best the evolution of the sword [7,8]. This is based on a traditional phylogeny of the genus that places the platies basal to the more derived swordtails and laboratory choice experiments that showed that platy females prefer heterospecific sworded males over their non-sworded conspecific males [7,20]. The traditional phylogenetic hypothesis therefore suggested that the females’ preference for the sword arose before the trait itself, and hence, the female preference might have driven the subsequent evolution of the males’ trait. The pre-existing bias hypothesis relies on an explicit phylogenetic hypothesis and can therefore be tested [3,21].

However, recent molecular phylogenetic studies suggested that the swordless platy species may instead be more derived than the more basally-placed sworded lineages [11,22]. This tree topology called the applicability of the pre-existing bias hypothesis for the evolution of the sword into question since the reconstruction of the evolution of the sword based on the molecular phylogeny suggested that the sword originated in the ancestor of this genus and was lost repeatedly and independently during the evolutionary history of this genus [11,22]. This topology further suggests that the females’ bias for swords might have been retained in the derived, but non-sworded platyfish species [7,20,23]. But subsequent testing of female preferences for swords among poeciliid species outside the genus *Xiphophorus*, namely of *Priapella*, showed that females of these species also preferred sworded males [8]. Since *Priapella* is one of the closest genera to *Xiphophorus*[24] and *Priapella*, just as all other poeciliid males do not have swords, their females’ preference for swords, would tend to lend support again to the pre-existing bias hypothesis as the best explanation for the initial evolution of the sword.

Obviously, the correct phylogeny for the genus is important for the inferred history of the sword. Several previous studies have performed analyses of ancestral state reconstruction of the sword in the genus *Xiphophorus* to understand its evolutionary history and to test for the pre-existing bias hypothesis. Some studies differed from each other in terms of how the sword was scored since some species are polymorphic in length or coloration of the sword. This, as well as whether parsimony or maximum likelihood was used, could somewhat alter results of the ancestral state reconstruction [25-27]. Based on the molecular phylogeny “sworded” was inferred to be the ancestral condition for all *Xiphophorus* species when caudal extension (of any length) was considered a sword [26,27], whereas its ancestral state was inconsistent - when short extension was assigned to another state (i.e. protrusion) [26]. Wiens and Morris [27] argued that uncolored “protrusion” should not be scored as a sword since the pre-existing bias was demonstrated through female preference for colored caudal extension [7]. They also demonstrated that “swordless” is an ancestral condition in their parsimony analysis supporting the pre-existing bias for the evolutionary origin of the sword. However, a likelihood reconstruction using the same description of the sword (i.e., colored extension) favored by Basolo [7] and Wiens and Morris [27], again resulted in an uncertain ancestral state [25]. Since the evolution of female preferences for swords has become a textbook example for the pre-existing bias hypothesis we, therefore, revisited this issue here based on the most comprehensive phylogeny, so far, most comprehensive, both in terms of taxa and markers.

Many of the previous phylogenetic analyses of this genus have been conducted solely (or at least mostly) based on either mitochondrial or morphological characters and the recently described four new species (*X. kallmani*[28], *X. mayae*[29], *X. mixei* and *X. monticolus*[30]) were not included in any phylogenetic analysis so far. Since ancestral state reconstructions need to be performed based on the most comprehensive phylogeny using different sword descriptions and different reconstruction methods (e.g., parsimony and likelihood) to understand the origin of the sword more clearly we set out to do this here.

Hybridization has been claimed to be one of the major modes for the origin of new species in some evolutionary lineages [31-33] and natural hybridization events between distinct populations or closely related taxa have been reported in various plants and animal taxa (e.g., [32,34-39]). Introgressive hybridization has been observed also in some lineages of freshwater fishes, for instance, whitefish [40], Lake Tanganyikan cichlids [41,42] and cyprinid fish [37]. Hybrid speciation by comparison, has been documented only rarely [33]. The role of natural hybridization in speciation is still debated due to the general observation of decreased fitness and sterility of hybrids [31,43].

It turns out that *Xiphophorus* fish are an excellent model system for examining the role of hybridization in speciation since we previously discovered that one species of this genus might be of hybrid origin [11,22]. Discordance between different types of molecular markers is routinely recognized as evidence for hybridization events, and such discordance has been uncovered, for example, in flies [44], goats [45], leaf monkeys [46] and vipers [47]. Previously, Meyer *et al*. [11,22] found a discrepancy in the placement of the swordtail species, *X. clemenciae*, in mitochondrial versus nuclear marker based phylogenetic trees. *Xiphophorus clemenciae*, a southern swordtail, grouped with the southern swordtails in the nuclear phylogeny [11], but was assigned to the southern platyfish lineage in the mitochondrial phylogeny. Meyer *et al*. [11] suggested that *X. clemenciae* originated in a relatively ancient hybridization event between a swordless female platyfish from a geographically widespread lineage such as *X. maculatus*, and a similarly widespread southern swordtail species, such as *X. hellerii*. Additional lines of evidence, including laboratory mate choice trials, the intermediate length of the sword in *X. clemenciae* and artificially produced hybrids relative to the two putative close relatives of the parental species, *X. maculatus* and *X. hellerii*[11], further support the hypothesis of a hybrid origin of *X. clemenciae*. Interestingly, on-going hybridization has been reported to occur between the northern swordtails *X. malinche* and *X. birchmanni*[39,48] and hybrids can be produced under laboratory conditions for most species in this genus [49-52].

**Figure 1:**
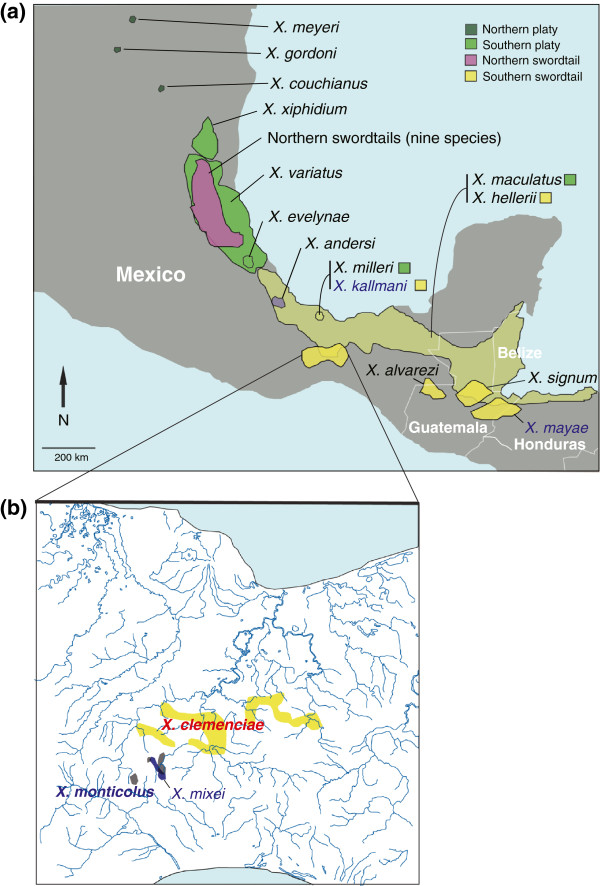
**Map of the distributions of *Xiphophorus *species. **(**a**) Geographical distributions of all described 26 species in the genus *Xiphophorus *including the four newly described species – *X. monticolus*, *X. mixei*, *X. kallmani *and *X. mayae *(colored in blue) and two species of a putatively hybrid origin, *X. monticolus *and *X. clemenciae *(in bold). (**b**) Geographical distributions of three species in the *clemenciae *clade (maps are modified from [2,58]).

Although the origin and evolution of the sword [7,20,26,53-56] and the role of hybridization in the genus *Xiphophorus*[2] have been addressed before, some of the phylogenetic relationships in this genus still remained uncertain. Traditionally, the genus *Xiphophorus* has been suggested to consist of four major lineages based on their geographical distributions and other phenotypic traits (i.e., northern platyfish, northern swordtails, southern platyfish and southern swordtails; Figure 1a) [2,11,21,22]. The monophyly and the relationships among those four lineages are not consistently supported in phylogenetic studies using molecular or combined molecular and morphological traits. For example, it has been difficult to assign *X. andersi*[57] to any specific lineage and inconsistent phylogenetic placements were found based on morphological characters and molecular based phylogenetic analyses [11,22]. *Xiphophorus andersi* has some platy as well as some swordtail features – it is an elongated – swordtail-like – species, but lacks the pronounced, colored ventral extension of the caudal fin. Also, geographical distributions of some species are inconsistent with those of other members of the lineages to which they were assigned; for example, a southern platyfish, *X. xiphidium* occurs further north than the northern swordtails [2] (see Figure 1a).

The northern swordtail lineage has received much attention from researchers because of its remarkable diversity in sexual and ecological traits [55,59-61], but the phylogenetic relationships among some of its nine described species remain incompletely resolved as well, hindering the interpretation of data in a phylogenetic context. Rauchenberger *et al*. [21] presented a comprehensive phylogeny using morphology, pigmentation and electrophoretic characters and suggested that there are three clades within this group – the *montezumae* clade (*X. nezahualcoyotl*, *X. continens* and *X. montezumae*), the *pygmaeus* clade (*X. nigrensis*, *X. multilineatus* and *X. pygmaeus*) and the *cortezi* clade (*X. cortezi*, *X. birchmanni* and *X. malinche*). However, these clades have not been consistently supported in subsequent phylogenetic studies using morphology, molecular data or a combination of those (e.g., pigmentation, allozyme, RAPD [Random Amplified Polymorphic DNA], mtDNA and nuclear DNA) [11,21,22,62-66].

Recently, four additional species have been described in this genus: *X. kallmani*, *X. mayae*, *X. mixei* and *X. monticolus* (Figures 1a, b). All these four new species are southern swordtails based on their geographical origins and phenotypic characteristics [2]. Yet, their molecular phylogenetic relationships to the other *Xiphophorus* species have not been examined so far.

Here, we conduct a comprehensive molecular phylogenetic analysis of the genus *Xiphophorus* that includes also these four newly described species. By using more informative nuclear markers, we aim to provide a better understanding of the phylogeny of this entire genus, its evolutionary history, and the evolution of the sword. We discovered that one of the newly described species, *X. monticolus*, is likely to have originated from an ancient hybridization event, as we found *X. clemenciae* to be the case before.

## Results

### Phylogenetic analyses

We reconstructed the phylogenetic relationships of the genus *Xiphophorus*, including four newly described species, using four different methods [i.e., BI (Bayesian Inference), ML (Maximum-Likelihood), NJ (Neighbor-Joining), MP (Maximum Parsimony)]. Two mitochondrial (cytochrome *b* and control region) and eleven nuclear loci [recombination activating gene 1 (Rag 1)/exon 3, tyrosine kinase (X-*src*), three non-coding flanking regions of the microsatellite loci (D2, D8 and T36) [11,22,67], guanine nucleotide-binding protein (G protein) subunit gamma13 (GNG 13), glucose-6-phosphate dehydrogenase (G6PD, 6^th^ intron), Uracil-DNA-glycosylase (UNG, 4^th^ intron), DNA polymerase beta (POLB, 7^th^ to 11^th^ intron), flap structure-specific endonuclease 1 (FEN1, 3^rd^ intron) and tumor protein p53 (TP53, 4^th^ intron)] were used for phylogenetic analyses. Since mitochondrial and nuclear DNA have different evolutionary histories, mitochondrial and nuclear phylogenetic trees were separately reconstructed. The total lengths of the aligned sequences used for the mitochondrial and nuclear phylogenies were 1239 bp and 7276 bp, respectively; of which 291 (218; without outgroup) nucleotide sites were variable and 192 (120) of those were parsimony informative for the mitochondrial loci, whereas 690 (499) nucleotide positions were variable and 412 (247) of those were informative for the nuclear loci. In addition, we reconstructed the phylogeny using a combination of the mitochondrial and nuclear data (8515 bp) to provide an overall view of evolutionary relationships of *Xiphophorus* using all data (Additional file 1). This combined tree showed nearly identical phylogenetic relationships among the major lineages (i.e., northern platyfish, northern swordtails, southern platyfish and southern swordtails) with the nuclear tree. Two species, *Priapella compressa* and *P. olmecae*, were selected as outgroups considering previously published phylogenies of the family Poeciliidae [24,68] as well as our recently reconstructed poeciliid phylogeny (Kang and Meyer, unpublished data). Both previous poeciliid phylogenies [24,68] independently support several different species as closely related taxa to *Xiphophorus*, although with very low support and conflicting relationships. Our recent poeciliid phylogeny (Kang and Meyer, unpublished data), which is based on several mitochondrial and nuclear DNA markers combined (7942 bp) and is the largest data set so far and provides support for the genera *Heterandria* and *Priapella* being the most closely related taxa to *Xiphophorus*; however, *Heterandria* showed a longer branch than *Priapella* in the phylogeny, which is also consistent with a recent RAD-marker based *Xiphophorus* phylogeny from the Meyer laboratory (Jones *et al*., in press). Genetic diversity indices and evolutionary models for each locus are shown in Table 1.

**Table 1:** Genetic diversity indices from two mitochondrial and eleven nuclear loci examined in this study

**Name**	**Locus**	**Nucleotides (bp)**	**Variable sites**	**Parsimony-informative sites**	***p*-distance**	**SE**	**Model of evolution**
**Nuclear**	Combined	**7276**	**690**	**412**	**0.017**	**0.001**	**TVM+G**
D2	Flanking region of the microsatellite loci D2	393	55	34	0.028	0.004	TIM3+G
D8	Flanking region of the microsatellite loci D8	516	59	37	0.019	0.003	TPM2uf+G
T36	Flanking region of the microsatellite loci T36	394	45	33	0.024	0.004	HKY
X-*src*	Tyrosine kinase	520	66	45	0.024	0.003	TVM+I
Rag1	Recombination activating gene	1574	64	40	0.007	0.001	TIM3+G
GNG13	Guanine nucleotide binding protein (G protein) subunit gamma 13 (1^st^ intron)	531	46	33	0.017	0.003	TPM2uf
G6PD	Glucose-6-phosphate dehydrogenase (6^th ^intron)	526	48	27	0.018	0.003	HKY+G
UNG	Uracil-DNA-glycosylase (4^th ^intron)	277	18	10	0.011	0.003	JC+G
POLB	DNA polymerase beta (7^th ^to 11^th ^intron)	672	43	22	0.01	0.002	TPM3uf+G
FEN1	Flap structure-specific endonuclease 1 (3^rd ^intron)	827	123	63	0.021	0.003	TPM3uf+G
TP53	Tumor protein p53 (4^th ^intron)	1046	123	68	0.024	0.003	TPM1uf+G
**mtDNA**	Combined	**1239**	**291**	**192**	**0.051**	**0.003**	**TPM1uf+I+G**
cyt*b*	Cytochrome *b*	360	112	67	0.059	0.006	TPM1uf+I+G
D-loop	Control region	879	179	125	0.047	0.004	TIM2+I+G

Nucleotide diversity (the average of *p*-distance between all the species) and standard error (SE) estimate were calculated using MEGA 4.0 [69]. Nucleotide diversity indicates estimates of average sequence divergence across all sequence-pairs. SE was estimated by a bootstrap procedure (1000 replicates). The best-fit evolutionary model was selected for each gene as well as for combined entire sequences of mitochondrial and nuclear genes using jModeltest under the Akaike Information Criterion [70].

### Mitochondrial phylogeny

**Figure 2:**
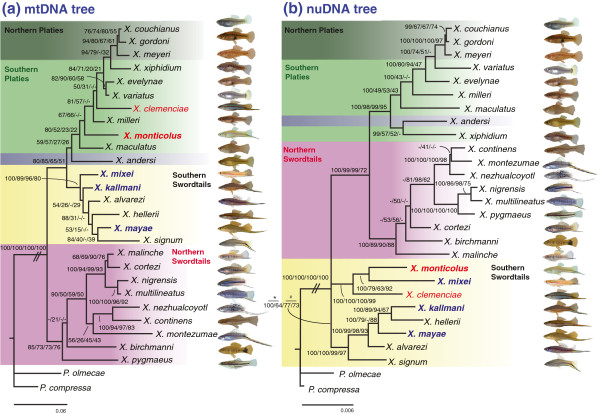
**Mitochondrial and nuclear phylogenies of all 26 *Xiphophorus *species. **The phylogenetic trees were constructed from (**a**) combined sequences of two mtDNA loci (1239 bp) (complete control region and a segment of the cytochrome *b *gene) and (**b**) combined sequences of eleven nuclear loci (7276 bp). We indicate with (*) and (#) the supporting values of monophyly and paraphyly of the southern swordtails respectively. Numbers indicate Bayesian posterior probabilities, Maximum-Likelihood, Neighbor-Joining and Maximum-Parsimony bootstrap values, respectively. The values of the branch length that was truncated are 0.447 (**a**) and 0.038 (**b**). The two hybrid origin species – *Xiphophorus monticolus*, one of the four newly described species, and *X. clemenciae* are highlighted in red and the three remaining new species, *X. mixei*, *X. kallmani *and *X. mayae *in blue. Some fish images were obtained from the *Xiphophorus *Genetic Stock Center (Texas) with permission.

The phylogeny based on the mtDNA markers placed the northern swordtails as the sister group to the clade formed by southern swordtails and platyfish (Figure 2a), which is consistent with previous mitochondrial phylogenies [11,22]. But the sister group relationship between the platyfish and the southern swordtails was supported by only moderate bootstrap values (51–85) in all phylogenetic estimations (i.e., BI, ML, NJ, MP) (Figure 2a).

Whereas *Xiphophorus monticolus*, although clearly phenotypically a southern swordtail, was placed with the southern platy group, the other three newly described species (*X. kallmani*, *X. mayae* and *X. mixei*) were placed in the southern swordtail clade. The monophyly based on mtDNA of the southern swordtails, except *X. clemenciae* and *X. monticolus*, was supported with high Bayesian posterior probabilities (100) and quite convincing bootstrap values (80–96), but the phylogenetic relationships within this southern swordtail group could not be resolved with high phylogenetic confidence.

The monophyly of the northern swordtail lineage was supported, albeit only with moderate bootstrap values (73–85), but the phylogenetic positions of *X. birchmanni* and *X. pygmaeus* were not consistent among the four different phylogenetic analysis methods. Overall, the recovered phylogeny of the 26 species of *Xiphophorus* was almost identical to our previous 22-taxa mtDNA-phylogeny [11].

### Nuclear phylogeny

The phylogeny based on eleven nuclear loci (see Table 1) provided good evidence for the monophyly of the platyfish plus *X. andersi* with high Bayesian posterior probabilities (100) and high bootstrap values (98) for maximum likeli-hood (Figure 2b). The monophyly of the northern platies was invariably strongly supported in all types of phylogenetic inferences, whereas the monophyly of the southern platyfish was not supported (Figure 2b).

The monophyly of the northern swordtails was strongly supported by all phylogenetic analyses (Figure 2b), whereas the southern swordtails were resolved as paraphyletic in some analyses. We found that the position of the two major lineages of swordtails in relation to the platies was differently resolved between the nuclear DNA and mtDNA phylogenies – and hasten to note, that the alternative topologies were relatively weakly supported only. Specifically, the platies (northern and southern) were more closely related to the southern swordtails than the northern swordtails in the mtDNA phylogeny, whereas the northern swordtail clade was identified as the sister group to the platies in the nuclear DNA phylogeny (Figure 2b).

Although the monophyly of the northern swordtails was strongly supported by all phylogenetic analyses (Figure 2b), within the northern swordtails, only two clades (the *montezumae* lineage and the *pygmaeus* lineage) were well-supported. Both sets of markers supported the monophyly of the northern swordtails, however, internal relationships were not clearly resolved in whole species phylogenies.

Contrary to the mtDNA-based tree, all four newly described species were grouped with previously recognized southern swordtail species [2,11]. Our data provide high support for both the *clemenciae* and *hellerii* clades (see also [30]) including all the newly described species in all phylogenetic methods (Figure 2b). Two of the newly described species, *X. mixei* and *X. monticolus*, group together with *X. clemenciae* (*clemenciae* clade), whereas the other two new species, *X. mayae* and *X. kallmani*, group with the remaining southern swordtails including *X. hellerii* (*hellerii* clade) (Figure 2b).

The analyses of the nuclear DNA data suggest that the *hellerii* clade is basal to all other swordtails and platies, and – tentatively – the *clemenciae* clade is the sister group to the northern swordtails plus the platies. While BI and ML (but with only 64% bootstrap support) methods suggest that the southern swordtails are paraphyletic, NJ and MP methods support their monophyly with bootstrap values of 77 and 73, respectively. We stress that the hypothesis of monophyly of the southern swordtails could not be rejected [*P* = 0.472, Approximately Unbiased (AU) test; *P* = 0.965, Shimodaira-Hasegawa (SH) test] and we (Jones *et al.,* in press) have a very large RADseq data set that also supports the monophyly of southern swordtails and their basal placement in the genus as sister to the platies+northern swordtails. Therefore, we continue to regard the monophyly of the southern swordtails to be more strongly supported. Our RADseq (restriction site-associated DNA sequencing) data set (Jones *et al*., in press) on this issue provides the strongest phylogenetic support yet for the monophyly of the southern swordtails based on a data set of about 66,000 SNPs. It remains an open issue why these four phylogenetic methods suggest a different basal node for the genus based on mtDNA and nuclear data sets.

### Discrepancy between mtDNA and nuclear DNA phylogenies: indication for a hybrid origin of *Xiphophorus monticolus*

Our extended phylogenetic analyses, including six new nuclear loci (3879 bp) and four new species, now provide evidence for the hypothesis of a hybrid origin of two *Xiphophorus* species, *X. clemenciae* and *X. monticolus*. Incongruence was found in the placement of both of these southern swordtail species between the mitochondrial and nuclear phylogenies (Figure 2) in that mitochondrially those two species were placed among the southern platies and, based on nuclear DNA sequences, these two species of southern swordtails were resolved to be part of the southern swordtail clade – a rather distant lineage of the genus they clearly belong to phenotypically. These results confirm the previously reported discrepancy in the placement of *X. clemenciae*[11,22] and suggest that an additional species, *X. monticolus*, also arose through similar mechanisms.

Therefore, the observed incongruence was further analyzed to determine whether the mtDNA phylogeny is indeed different from the nuclear tree with respect to the positions of *X. monticolus* and *X. clemenciae*. We compared both best mitochondrial and nuclear ML unconstrained trees (Figure 2) with their best ML constrained trees. In the mitochondrial data set, the best ML unconstrained tree (Figure 2a) was strongly favored in comparison to the constrained tree, which placed: 1) *X. monticolus* with *X. mixei* and 2) *X. monticolus* with *X. mixei* and *X. clemenciae*, similar to the nuclear tree (unconstrained tree; *P* = 1.00, constrained tree; *P* < 0.05, AU and SH tests; Table 2) [71]. In the nuclear data set, the best ML unconstrained tree (Figure 2b) was significantly better at “explaining” the nuclear data set than the best ML constrained tree, which assigned to 1) *X. monticolus* with the platies or 2) *X. monticolus* with the platies, apart from *X. maculatus* (unconstrained tree; *P* = 1.00, constrained tree; *P* < 0.001, AU and SH tests; Table 2). Both phylogenetic hypotheses that place *X. clemenciae* and *X. monticolus* with the platies in the nuclear phylogeny, and that group these species with the southern swordtails in the mitochondrial phylogeny were strongly rejected (*P* < 0.05, AU and SH tests; Table 2).

**Table 2:** Comparison of the best ML unconstrained trees with the constrained best ML trees of both mitochondrial and nuclear phylogenies

**Mitochondrial DNA**	**AU (SE)**	**SH (SE)**	**Nuclear DNA**	**AU (SE)**	**SH (SE)**
Unconstraint best ML tree (Figure 2a)	0.992 (0.001)	0.989 (0.001)	Unconstraint best ML tree (Figure 2b)	1 (0)	1(0)
Constraint best ML tree (*X. monticolus *with *X. mixei*)	0.010 (0.002)	0.022 (0.001)	Constraint best ML tree (*X. monticolus *with platies)	7.00E-005(0)	1.00E-04(0)
Constraint best ML tree (*X. monticolus *with *X. mixei* and *X. clemenciae*)	0.001 (0)	0.001 (0)	Constraint best ML tree (*X. monticolus *with platies except *X. maculatus*)	2.00E-058(0)	0(0)

*P*-values were estimated by AU [Approximately Unbiased] and SH [Shimodaira-Hasegawa] tests implemented in CONSEL [72] (SE: standard error).

If *X. monticolus* arose by hybridization*,* closely related extant taxa might be genetically close to the putative maternal and paternal species. In the mitochondrial phylogeny, *X. monticolus* is closely related to platies such as *X. evelynae*, *X. variatus*, *X. milleri* and *X. maculatus* with 1.9%, 2.3%, 2.5% and 2.9% sequence divergence, respectively. In the nuclear phylogeny, *X. monticolus* was grouped however, with *X. clemenciae* and *X. mixei* (with 0.9% and 1.1% of sequence divergence only, respectively). The taxa most genetically similar to *X. monticolus*, apart from the *clemenciae* clade (*X. clemenciae*, *X. mixei* and *X. monticolus*), in the nuclear tree were *X. mayae* and *X. signum* with 1.1% and 1.2% sequence divergence. These data provide some hints as to the timing, species identity of the maternal and paternal lineages, and phylogeography of the hybridization event (see below).

### Northern swordtail phylogeny

**Figure 3:**
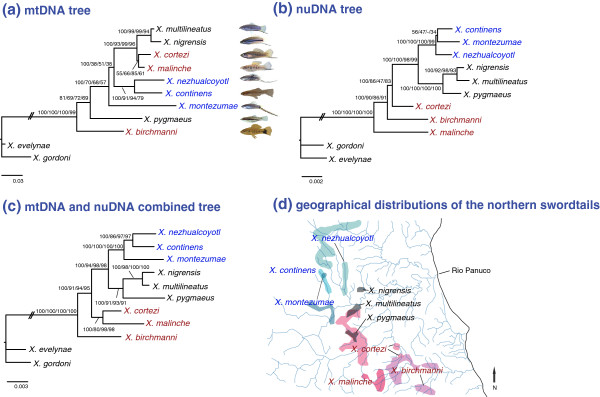
**Mitochondrial and nuclear phylogenies of the nine northern swordtail species.** The phylogenetic trees were constructed from (**a**) combined sequences of two mtDNA loci (1235 bp) (complete control region and a segment of the cytochrome *b* gene), (**b**) combined sequences of eleven nuclear loci (7073 bp), and (**c**) combined sequences of two mitochondrial and eleven nuclear loci (8308 bp). Numbers indicate Bayesian posterior probabilities, Maximum-Likelihood, Neighbor-Joining and Maximum-Parsimony bootstrap values, respectively. The values of the branch length that was truncated are 0.115 (**a**), 0.007 (**b**) and 0.012 (**c**). Patterns of the geographical distributions of the nine species in the northern swordtails are shown (**d**) (map is modified from [2]). Species in the same clades inferred by [21] are shown in same color.

The relationships among the nine northern swordtail species differed more between the mtDNA and nuclear DNA phylogenies (Figure 2). Because species of the northern swordtail lineage are used by several laboratories for behavioral ecological work and as model for study of evolutionary questions, we, therefore, conducted additional analyses on the northern swordtails only - based on the nuclear and mitochondrial data sets separately and also combined both data sets (Figure 3) – in an effort to resolve the phylogenetic relationships among the species in this lineage. For these analyses, two platyfish species (*X. evelynae* and *X. gordoni*) were used as outgroups (Figure 3). Aligned nucleotide sequences of the mitochondrial loci contained 151 variable sites and 85 of those were parsimony informative with 0.041 [SE (Standard Error) = 0.003] of average *p*-distance, whereas the nuclear loci exhibited 245 variable sites and 110 of those were informative with 0.01 (SE = 0.001). The best-fit evolutionary models chosen (jModeltest 0.1.1, [70]) for the mitochondrial and nuclear loci were TPM1uf+I+G and TPM1uf+G, respectively. The combined mitochondrial and nuclear alignments contained 396 variable sites and 195 of these were parsimony informative with 0.015 (SE = 0.001) of average *p*-distance and TrN+G was determined as the best-fit evolutionary model.

Analyses of the northern swordtails based on the nuclear and mitochondrial separate data sets (Figures 3a, b) revealed almost identical tree topologies compared to the whole nuclear and mitochondrial phylogenies with all species (Figures 2a, b). Nevertheless, those analyses provided much higher bootstrap values and all four different phylogenetic estimations (BI, ML, NJ and MP) agree on the majority of nodes. In the nuclear phylogeny (Figure 3b), we recovered the *pygmaeus* and *montezumae* clades, but not the *cortezi* clade, which is consistent with several previous studies using different markers and morphological characters [11,21,64-66]. The mitochondrial and nuclear combined data set (Figure 3c) showed similar phylogenetic relationships to the nuclear phylogeny, and still could not recover the *cortezi* clade. In the mitochondrial phylogeny (Figure 3a) however, some phylogenetic relationships are incongruent with the nuclear phylogeny. For example, two strongly supported clades (*X. montezumae* and *X. pygmaeus* clades) in the nuclear phylogeny were not recovered in the mtDNA analyses. Here *X. birchmanni* might be basal to all other northern swordtails, but this remains unresolved in the nuclear phylogeny. The clade of *X. nigrensis* and *X. multilineatus*, which was strongly supported in our mtDNA, nuclear and combined phylogenetic analyses, and also previous studies [11,21,65], is grouped with *X. cortezi* and *X. malinche* in the mitochondrial DNA phylogeny, whereas it is more closely related to *X. pygmaeus* in the nuclear DNA phylogeny. These better resolved mitochondrial and nuclear phylogenetic analyses clearly show many incongruent phylogenetic positions between two types of molecular marker based phylogenies in the northern swordtails.

### Para- or monophyly of the southern swordtails

We found conflicting support for monophyly (100% Bayesian and 64% ML) or paraphyly (77% NJ and 73% MP) of the southern swordtails based on the four phylogenetic methods used for the nuclear markers (Figure 2b and see combined tree Additional file 1). But, the hypothesis of monophyly could not be rejected (see above) and was very strongly supported by our unpublished RADseq data set (Jones *et al*., in press). To further investigate the phylogenetic relationships among the southern swordtails, we compared the topology of trees constructed using each gene individually (Additional file 2). Two classes of genes suggest different evolutionary hypotheses regarding monophyletic or paraphyletic relationship of the *clemenciae* and *hellerii* clades. Seven nuclear loci (D8, X-*src*, Rag1, GNG13, G6PD, POLB and FEN1) support a paraphyletic relationship of the southern swordtails, but three loci (UNG, TP53, T36) show monophyly and one locus (D2) could not show their relationship clearly (Additional file 2). Further phylogenetic analyses based on the combined set of those seven markers inferred the paraphyly (Additional file 3a) and four markers (UNG, TP53, T36 and D2) supported the monophyly of the southern swordtails (Additional file 3b) with higher bootstrap values than when the eleven markers were combined. Clearly, there are partially conflicting phylogenetic signals in this set of eleven nuclear markers. Overall, our data does not strongly discriminate between, or statistically reject, monophyly or paraphyly of the southern swordtails. Further studies are required to determine the evolutionary history of the southern swordtails, yet our RADseq markers and strongly support the monophyly of the southern swordtail clade and their basal placement (Figure 2b) in the genus.

### Ancestral state reconstructions of the sword

We constructed the ancestral state of the sword using our nuclear marker based phylogenetic tree that places the southern swordtail clade basal in the genus.

Several factors were considered in selecting a nuclear tree topology for the ancestral state construction of the sword. For the platies, we implemented a tree topology where *X. andersi* is basal to all platies, a topology also inferred by a previous study [11]. For the northern swordtail group, a tree topology based on the combined mtDNA and nuclear data sets was applied since it was always strongly supported at every node using different phylogenetic methods (Figure 3c). Monophyly of the southern swordtails was implemented (see above).

**Figure 4:**
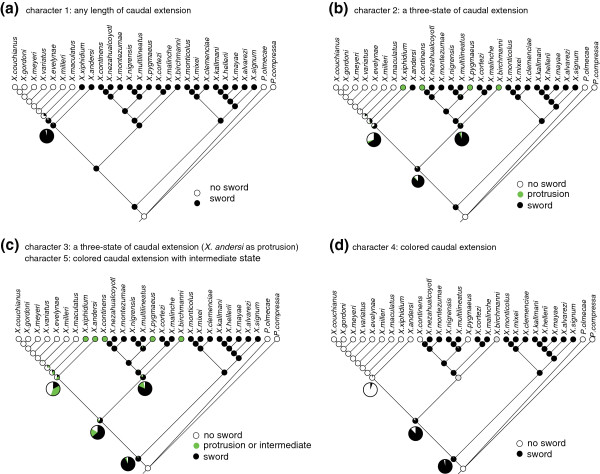
**Maximum-likelihood reconstructions for the ancestral state of the sword in the genus *Xiphophorus*. **Five different characters were mapped onto the nuclear tree: (**a**) a two-state character of sword extension (character 1), (**b**) a three-state character of sword extension (no sword, protrusion and sword; *X. andersi *was coded as a sworded species, character 2), (**c**) a three-state character of sword extension (*X. andersi *was coded as a species with protrusion, character 3) [26] and colored caudal extension with intermediate state (character 5), and (**d**) a two-state colored caudal extension (*X. birchmanni *was coded unknown, character 4) [27]. ML analyses of the character states, the colored caudal extension with polymorphic state (character 6), coloration (character 7) and the ventral black margin (character 8) of the sword, were not performed since Mesquite does not allow performing ML calculations on the characters that are polymorphic in some taxa. Each circle on the nodes represents character state (black filled circles: sword; green circles: protrusion or intermediate; empty circles: no sword; grey circles: unknown).

The definition of the sword and composite traits has long been debated and, on occasion differently interpreted in various species descriptions [16,21,26,57]. Also previous studies of ancestral state reconstruction have shown inconsistent ancestral states for the sword, depending on both how the sword was scored and which reconstruction methods were used [25-27]. Here, a comprehensive set of reconstructions of ancestral state was conducted by applying different sets of scoring of the sword (Figure 4 and Additional file 4) and by including other sets of sword traits (coloration, ventral black margin; Additional file 5), using both maximum likelihood (Figure 4 and Additional file 6) and parsimony approaches (Additional files 4, 5). Detailed descriptions of the sword characters and sword scorings are given in the Methods (see below).

In our maximum-likelihood analyses, the possession of a sword was always recovered as the ancestral state at the base of the genus *Xiphophorus* with high proportional-likelihood (0.921-0.996) in all character states (Figure 4, Additional file 6). However, in the parsimony analyses the ancestral states were not consistent since the ancestral state of the sword changed based on 1) how we treated the transition state of swords as ordered or unordered (i.e., protrusion is an intermediated step from ‘no sword’ to ‘sword’ states) and 2) how we defined the sword (i.e., only in terms of length or whether a sword also has to be a colored extension) (Additional file 4). The ancestral state of the sword was more ambiguous when the sword was coded as colored caudal extension (characters 4–6, see Additional file 4) rather than if only length of an extension of the caudal fin was considered to be a “sword” (characters 1–3, Additional file 4). However, clearly, none of the characters in any analysis ever supported “swordless” as the ancestral condition in the genus *Xiphophorus*. Other sword traits (coloration, character 7; ventral black margin, character 8, Additional file 5) could not be reconstructed clearly because the absence or presence of these state of those traits was inferred to be equally parsimonious.

The sword clearly was lost at least once (more likely more than once, depending on the character state coding and reconstruction method) in this genus, but the repeated evolution of the sword could not be inferred strongly (Figure 4). It was lost once in the southern platy lineage when the sword was considered as a two-state character of sword extension (no sword, sword: character 1, Figure 4a). The sword appears to have “shortened” four times from a sword to short protrusion (or intermediate) during the evolution of this genus and it was inferred to have been lost completely once in the platy lineage under the definition of the sword as a three-state character of sword extension (no sword, protrusion or intermediate, and sword: characters 2, 3, 5, see Figures 4b, c). Another two-state character of the sword, the colored extension (character 4, Figure 4d), was lost twice in the northern swordtail lineage and once in the platyfish. These results suggest that the ancestral state at the root of the genus is “sworded”. Furthermore, loss of sword or colored sword traits occurs much more frequently than their gain.

## Discussion

### Hybrid origin of *Xiphophorus monticolus*

We show that one of the recently described *Xiphophorus* species, *X. monticolus*, is likely to have arisen through an hybridization event, similar to what has been shown before for *X. clemenciae*[11,22]. The hybrid origins of the species, *X. monticolus* and *X. clemenciae*, are the most likely explanation for several reasons including, but not limited to their incongruous placement in the mtDNA and nDNA trees (Figure 2). However, we cannot completely rule out that the discrepancy between phylogenetic placements based on different marker types does not result from other biological processes such as incomplete lineage sorting (ILS) [73,74]. However, we regard this as a much less plausible hypothesis. If ILS were the explanation for this discrepancy, a particular locus or group of loci would be expected to lead to different phylogenetic relationships from others. To evaluate whether particular loci have a particularly strong effect on the phylogenetic results, eleven separate nuclear trees were reconstructed by subtracting one locus at a time. Yet, we did not find evidence for strong locus-specific phylogenetic relationships among all the eleven independent phylogenies analyzed with regard to the positions of the two hybrid species. Hybridizations among several species of *Xiphophorus* have been observed in the wild [2] and laboratory-hybrids can be produced for most species of *Xiphophorus*[49-52]. All of these observations suggest that hybrid origins of species in this genus are feasible and appear to best explain the origin of these two species in this genus.

If *X. monticolus* and *X. clemenciae* indeed arose by hybridization it would be of great interest to know where and when this hybridization took place and which species are likely to be the parental lineages. We would like to point out that the sword indices (sword length / standard length) of *X. clemenciae* and *X. monticolus* are identical (0.25 for both species) whereas it is 0.06 for *X. mixei*[30], the closest relative of both *X. monticolus* and *X. clemenciae* (Figure 2b) [30]. The high similarity in sword length and gonopodium (male modified anal fin for internal fertilization) structures particularly in ray 4 or 5 between *X. clemenciae* and *X. monticolus* might further suggest that both species arose from hybridization between a platyfish and the same paternal species.

In the case of *X. clemenciae*, the discrepancy in the phylogenetic position was parsimoniously explained by an ancient hybridization event between a swordless platyfish, such as *X. maculatus* (or *X. milleri*)*,* as the maternal species, and a sworded southern swordtail as the paternal species, such as *X. hellerii* in the previous studies [11,22,58]. Meyer *et al*. [11] proposed that relatives of *X. maculatus* and *X. hellerii* might be parental species for *X. clemenciae* because those two species are closely related to *X. clemenciae* (in mtDNA and nuclear DNA, respectively) and have wide and overlapping geographic distributions. In addition, Meyer *et al*. [11] found that laboratory crosses between female *X. maculatus* and male *X. hellerii* resulted in hybrids with intermediate sword lengths. They argued that because *X. clemenciae* has an intermediate sword length between *X. maculatus* (no sword) and *X. hellerii* (sword), *X. clemenciae* may have arisen from two species such as those.

Here we suggest that *X. monticolus* may also have originated from a hybridization event between a southern platy and a southern swordtail, and again species such as *X. maculatus* (or an ancestor of *X. maculatus*) may be the maternal species and *X. hellerii* or alternatively *X. mixei* (or an ancestor of those species) may be paternal species. Although other platies (e.g., *X. variatus*, *X. evelynae* and *X. milleri*) are genetically more closely related to *X. monticolus* (Figure 2a), as is the case of *X. clemenciae, X. maculatus* (or its ancestor) is deemed to be the more likely maternal species because of its wider geographic distribution (see Figure 1). We caution that, of course, the current distributions may not necessarily resemble ancient distributions and therefore inferring parental lineages using current geographical information may not be reliable. Additionally, our comparative morphological study of *Xiphophorus* species revealed that *X. maculatus* has more similar gonopodial structures to the species in the *clemenciae* clade (e.g., *X. mixei*) than other platies (Jones *et al*., unpublished data). If the gonopodial lock-and-key hypothesis applies, i.e. that genital morphology may prevent mating between different species [75], similar gonopodial structures between maternal (*X. maculatus*) and paternal species (*X. mixei*) might also lend further support to *X. maculatus* as a potential maternal species. Our favored hybridization scenario involves repeated backcrossing of hybrid females into the parental species with the longer sword which would explain that in terms of nuclear genes the hybrid species much more resemble their paternal species, as is clear shown in the nuclear DNA trees.

A *Xiphophorus mixei*-like fish is a likely alternative paternal lineage because it is the most closely related species to *X. monticolus* and *X. clemenciae* (Figure 2b) and the monophyly of *“clemenciae* clade” was strongly supported both phylogenetically and morphologically (Figure 2b, [30]). The three species in the *clemenciae* clade (*X. clemenciae*, *X. mixei* and *X. monticolus*) all have orange lateral stripes that are produced by carotenoid pigments, whereas members of the *hellerii* clade exhibit red stripes where the pigment is produced by drosopterin [76]. The spots (in red for *X. clemenciae* and in black for *X. mixei* and *X. monticolus*) in the proximal portion of the caudal fin in adult males were only detected in the *clemenciae* clade, but not in any other species in the genus *Xiphophorus*[30]. The species within each of the *clemenciae* and *hellerii* clades have more similar gonopodial structures to one another [30], in particular, the distal part of the gonopodia, including hook shape, spine numbers and slightly anteriorly angled tip of the ramus is more similar between species in the *clemenciae* group in comparison to species in the *hellerii* group [30]. Furthermore, an ongoing comparative study of the gonopodium of the genus *Xiphophorus* further supports the hypothesis that the distal structures of *X. mixei* are similar to both species of hybrid origin in the *clemenciae* clade than any other species in the *hellerii* clade (Jones *et al*., unpublished data).

*Xiphophorus monticolus* may have arisen from a “local” hybridization event since it is restricted geographically to headwater streams of the Rio Jaltepec, a major Rio Coatzacoalcos tributary, in Oaxaca, Mexico [30] (Figure 1b). Hence, *X. monticolus*, similar to what we believe to be the case for *X. clemenciae* as well, is likely to have arisen from a hybridization event at a single locality. Thus, genetic similarity, shared morphological traits, and their sympatric distributions (Figure 1b) further support the hypothesis of a hybridization event between the species related to *X. maculatus* and *X. mixei*.

Combined all phylogenetic, phenotypic and distribution data point towards *X. mixei* as a likely paternal lineage for the two independent hybridization events within the *X. clemenciae* clade. Further genetic studies of *X. mixei* are warranted to uncover more genetic traces of this species that might support the hypothesis that it was involved in these hybridization/speciation events. For example, distinct genetic mechanisms such as hemi-clonal lineages found in *Poeciliopsis monacha* may be analogous to the genesis of new hybrid species in the genus *Xiphophorus*[77]. *Poeciliopsis monacha* has an all-female system of reproduction, where females produce hybridogenic progeny that carry the maternal genome of *P. monacha* and replace it with a paternal nuclear genome of sexually reproducing species (e.g., *P. lucida*) in each generation [77]. Further research on ecological and behavioral aspects of *X. mixei* (e.g., female preference for the sword or other sexually selected traits) might provide interesting insights into the role of this species in the hybrid origin of its two closest relatives.

### Phylogenetic relationships among the northern swordtails

Combined mitochondrial and nuclear data (Figure 3c) provide a more resolved phylogeny compared to previous phylogenetic analyses. However, these results should still be regarded as tentative. For instance, the monophyly and relationships within the *coterzi* clade, and their relationships to other lineages remain unresolved, as examined in several previous investigations [11,62,63,65,66].

Many incongruent relationships identified by the two types of marker-based phylogenies might provide a hint for another putative hybrid origin from the northern swordtail species. For example, the mitochondrial data set indicates a well-supported sistergroup relationship between *X. nezahualcoyotl* (long sword) and *X. continens* (protrusion), whereas the nuclear DNA set suggests that *X. nezahualcoyotl* could be closely related to *X. montezumae* (long sword). However, the nuclear tree could not provide strong support for the grouping of *X. nezahualcoyotl* with *X. montezumae*, so further analyses using additional nuclear markers are required to confirm this relationship. If this relationship is supported by future analyses, as it actually is by our RADseq data (Jones *et al.,* in press), then it is likely that *X. nezahualcoyotl* might be of hybrid origin as well, on the basis of the incongruence of mitochondrial and nuclear trees. Further support for this hypothesis is provided by the notable morphological differences between the sister species *X. nezahualcoyotl* (long sword) and *X. continens* (short sword) in the mitochondrial tree (Figure 3a). This might suggests that hybrid speciation is not that uncommon within the genus *Xiphophorus* with hybrid origins for two of the 26 *Xiphophorus* species. Further studies are required to investigate the prevalence of hybrid origins and its overall role in speciation within the family Poeciliidae. However, several natural hybrid zones have been reported for some species-pairs (e.g., *X. birchmanni* – *X. malinche*) in the northern swordtail clade [39,48,63]. Possibly some specimens in this group might have been collected from hybrid zones, although we lack detailed information about the sampling localities for some specimens of this study (Additional file 7). Therefore, phylogenetic placements of those species need to be interpreted with caution.

### Evolutionary history of the sexually selected trait: the sword

The (repeated) loss of the most conspicuous aspects of the sword - its length and its coloration (Figure 4) - might suggest that the selective forces of natural selection repeatedly won over the forces of sexual selection that would tend to favor more obvious and exaggerated traits. The evolution of the sword clearly shows periods of reversals where a conspicuous long sword secondarily became less conspicuous and or shorter or was lost completely as at the origin of the platyfish. In future work it would be interested to investigate under what environmental conditions or biotic conditions natural selection might act most strongly against conspicuous and long swords. Several abiotic and biotic factors might play a role: (a) flow velocity of streams as longer sworded males would be expected to be at a hydrodynamic disadvantage, particularly in faster flowing streams, (b) water clarity and thereby visibility to females, but also predators, might also tend to select against males with particularly colorful or contrast swords (black strip), (c) particularly if the abundance of predators, in the water or from the air, that hunt visually or whose capture method might select against males with particularly long swords, that would tend to have a slower fast-start performance, is high.

The ancestral state of the sword remained disputed [16,25-27]. Previous character state reconstructions, based on the parsimony method [26], illustrated that the ancestral state reconstruction of the sword varied, depending on how the sword was categorized (i.e., no sword, protrusion and sword), and whether the transition between the two states (i.e., swordless and sworded) was treated as ordered or unordered. The use of likelihood reconstruction methods has similarly not provided a clear reconstruction of the ancestral state of the sword [25]. In the latter analysis the authors suggested two possible factors that might lead to uncertainty. One is that the “sword” was coded as a single trait with only two character states (i.e., no sword and sword) instead of three character states (i.e., no sword, protrusion, and sword). The other is possibly a higher rate of changes in sword character states in *Priapella* (a genus that has no sword at, just as all other poeciliids outside the genus *Xiphophorus*) than in *Xiphophorus* although the latter clade may have higher transition rate [25]. The ambiguity of the ancestral states in previous studies might also be affected by incomplete phylogenies since all previous analyses on the reconstruction of the evolution of the sword were conducted based on morphology-based phylogenies, where platies are basal to swordtails, or mtDNA-based trees where the four new species could not yet be included [16,25-27]. Mitochondrial phylogenies in particular were certainly misleading due to the incorrect placement of *X. clemenciae* among the platies as well as uncertainty about the topology of the tree in the deepest nodes and among the northern swordtails.

Our trait reconstructions using likelihood analyses covering all plausible sword scorings that have been used in several previous studies, implemented based on the nuclear marker based tree, show a consistent ancestral state, regardless of the different definitions of what constitutes the composite trait “sword”. These results clearly indicate that the sword very likely originated in the common ancestral lineage of the entire genus *Xiphophorus*, and that the sword has been lost completely (no sword) or partially (protrusion or intermediate) in different lineages independently and repeatedly (see above) (Figure 4).

By comparison, the parsimony analyses still showed ambiguous ancestral states under the three character states (character 3, unordered; character 4; character 5, unordered) (see Additional file 4) although the same character state codings as in the likelihood method were applied and both sets of analyses used the identical comprehensive nuclear phylogeny. Of course, the maximum parsimony method reconstructs the ancestral state that requires the smallest number of state changes. It has been suggested that ecological traits are prone to being biased because of the assumption of low rate of character changes or a stochastic element in parsimony analysis itself [25]. The ambiguous state reconstruction might therefore result from the method *per se* because changes in the characteristics of the sword are, apparently not that rare in *Xiphophorus*. Nevertheless, “swordless” was not found to be the ancestral state for the genus based on the parsimony analyses (Additional file 4).

Our analyses further suggest that the evolution of the sword is tending towards a reduction in length or even complete loss of the trait rather than secondary gains. Although the evolutionary origin of swords through female preference has been a central focus in the literature [7,8,11,16,20,22,23], little attention has been paid to the ‘loss’ or ‘reduction’ of the sword or other characteristics of the sword such as its coloration or the black stripe. The brightly coloration of sword will increase the conspicuousness of its bearers not only to females, but also to predators and therefore be disadvantageous for survival [78]. Additionally, increased sword length is costly for fast start (escape) and endurance swimming [79]. If female preference is a major driver for the evolution of the sword, a change or loss of female preference might also equally affect the loss of the sword traits (i.e., length or coloration), or since female preferences for the sword seem to have been retained in species, such as platies, in which their males had lost their swords, natural rather than sexual selection might have played a decisive role in that [22] (and see above). Alterations in female preference for the sword have been reported – for example, females prefer swordless conspecifics over sworded heterospecific males in *X. birchmanni*[80], and a change in female preference for the sword has been shown also for *X. nigrensis*[81,82].

If changes in female preference for the sword have indeed occurred frequently in response to different/changing environments or other factors impacting on mate choice, then our hypothesis that *X. mixei* constitutes the closest relative to the putative paternal lineage of *X. monticolus* (given that *X. mixei* has a relatively short sword) would gain further plausibility. This still needs to be tested in *X. mixei* females. It is also interesting to note that *X. mixei*, with its very short sword, is consistently nested within the *clemenciae* clade. So, despite being genetically closest to the hybrid species, and the putative “parental” lineage, *X. mixei*’s sword is very different from that of both *X. clemenciae* and *X. monticolus*. This observation, again, supports the notion that sword evolution can be fast and the phenotype is labile.

Future studies on the evolutionary relationships among *Xiphophorus* species would benefit from the inclusion of alternative outgroups since distant outgroups can possibly generate reconstruction artifacts. A robust phylogeny of the entire family Poeciliidae would help to clarify which species or genera are most closely related to the genus *Xiphophorus*. Further studies on ecological and behavioral features affecting the evolution of particular sword traits and the genetic compositions of hybrid species will help to unravel the origin of the repeated ancient hybridization events in the genus *Xiphophorus*. In a broader context, our study provides information on the long-standing controversy over the role and significance of hybridization in contributing to speciation and evolution in animals.

## Conclusions

In this study, we reconstructed the phylogenetic relationships of all known 26 species in the genus *Xiphophorus* using eleven nuclear loci and two mitochondrial loci. We show that an additional (to the previously known *X. clemenciae*) southern swordtail species, *Xiphophorus monticolus,* is likely to have arisen through hybridization. Our mitochondrial and nuclear marker based phylogenetic analyses showed discordance in the position of this species, similar to previous analyses of *X. clemenciae.* The other three other recently described species - *X. mixei*, *X. kallmani* and *X. mayae* - group together with the southern swordtail species in both sets of trees. Among the northern swordtails, while the monophyly of the *montezumae* and *pygmaeus* clades was strongly supported in the nuclear phylogeny, our data also suggest incongruent phylogenetic relationships between two types of marker-based trees. The ancestral state reconstruction of the sword, in particular using maximum likelihood approaches, strongly suggests that the common ancestor of the genus *Xiphophorus* already possessed a sword and that it was lost completely secondarily again in the derived platy lineage of the genus. Our complete molecular phylogeny of the genus *Xiphophorus* provides a comprehensive phylogenetic framework within which the results of all studies on this genus can now be interpreted.

## Methods

### Taxon sampling

Seventy-three individuals from all described species of the genus *Xiphophorus* (26 species) including 16 specimens from the recently described four species (*X. kallmani*, *X. mayae*, *X. mixei* and *X. monticolus*) were used. *Xiphophorus mixei* and *X. monticolus* were obtained from the *Xiphophorus* Genetic Stock Center (http://www.xiphophorus.txstate.edu, San Marcos, TX, USA). Two outgroup species, *Priapella compressa* and *P. olmecae*[24], were chosen based on our recently reconstructed a phylogeny for the poeciliids using several mitochondrial and nuclear DNA markers combined (7942 bp). The genera *Heterandria* and then *Priapella* were found to be the most closely related taxa to *Xiphophorus*, but *Heterandria* species showed a longer branch than *Priapella* species (Kang and Meyer, unpublished data). Sampling localities and detailed information about each of the samples are shown in Additional file 7 (lack of locality information is also indicated as ‘unknown’).

### DNA extraction, PCR amplification and sequencing

Total genomic DNA was extracted from fin clips or tissues preserved in ethanol using a Qiagen DNeasy tissue kit (Qiagen). Ethanol preserved samples were incubated in TE9 buffer (500 mM Tris, 20 mM EDTA, 10 mM NaCl, pH 9.0) overnight to improve the extraction efficiency by eliminating an excess of ethanol [83]. Polymerase chain reaction (PCR) was performed in 15 μl volumes containing 1x PCR buffer, 200 μM each dNTP, 1.5 mM MgCl_2,_ 0.6 μM each primer and 1U of High fidelity *Taq* polymerase (Fermentas).

We used published primers of the mitochondrial control region, cytochrome *b*, Rag1/exon 3, X-*src* and three non-coding flanking regions of the microsatellite loci (D2, D8 and T36) to obtain the sequences for the four newly described species. Sequences of these seven loci for the 22 remaining species were acquired from GenBank (see below). Six primer-pairs were newly designed to amplify the intronic regions of nuclear loci: GNG 13, G6PD (6^th^ intron), UNG (4^th^ intron), POLB (7^th^ to 11^th^ intron), FEN1 (3^rd^ intron) and TP53 (4^th^ intron) for all 26 species (See Table 1). For the GNG 13 locus, degenerate primers were designed based on the conserved exon flanking regions from five teleost species (zebrafish, medaka, fugu, tetraodon and stickleback) and the other five primer-pairs were developed using *X. maculatus* genomic sequences (which were provided by Ron Walter, *Xiphophorus* Genetic Stock Center, Texas State University, USA). DNA sequences of these new six primer-pairs and PCR conditions are provided in Additional file 8.

PCR was carried out using appropriate conditions for each primer pair, for example, 94°C for 3 min, 35 cycles at 94°C for 30 sec, 42-60°C for 30 sec and 72°C for 1-1min 30 sec. A final extension step at 72°C (7 min) was conducted. PCR products were checked on 1.5 % agarose gels, and then incubated at 37°C for 15 min and 85°C for 15 min with Exonuclease I and Shrimp Alkaline phosphatase (Fermentas), respectively in order to purify the PCR products [84]. The purified mtDNA and nuclear DNA fragments were subject to direct sequencing in the forward and reverse directions using the same forward and reverse primers as in the PCR and the BigDye Terminator 3.1 Cycle Sequencing Ready Reaction Kit (Applied Biosystems). All DNA sequencing reactions were run on a 3130*xl* DNA Analyzer (Applied Biosystems) and analyzed with ABI PRISM DNA Sequencing Analysis Software version 5.3.1. Both forward and reverse strands were sequenced for accuracy in each individual.

### Phylogenetic analyses

DNA sequences were analyzed from all 26 species plus two outgroup species *Priapella compressa* and *P. olmecae*. Two combined mitochondrial gene sequences (1239 bp) and eleven combined nuclear gene sequences (7276 bp) were used separately in the phylogenetic analyses. In addition, a combined mitochondrial and nuclear phylogeny (8515 bp) was reconstructed.

For the two mitochondrial loci, sequences of cytochrome *b* (360 bp) and control region (879 bp) were acquired for the four new species and were combined with published sequences for the 22 remaining species plus two outgroup species deposited in GenBank [11,22]. The total length of sequences, the number of variable sites, parsimony informative sites, and the nucleotide diversity of each mitochondrial and nuclear locus are shown in Table 1.

For the eleven nuclear loci, previously published sequence data from 22 species and the two outgroup species were used for five nuclear loci (Rag1/exon 3: 1574 bp, X-*src*: 520 bp, D2: 393 bp, D8: 516 bp and T36: 394 bp) [11,22], and new sequence data for the four newly described species were determined for these loci. Our newly developed six intron-makers were amplified for all 26 species and the two outgroup species. The sequences of the intronic regions of the genes, GNG13 (531 bp), G6PD (526 bp), UNG (277 bp), POLB (672 bp), FEN1 (827 bp) and TP53 (1046 bp) were combined with the previously published sequences of the other five loci in an effort to construct a nuclear marker phylogeny.

In addition to the phylogenetic analysis of all the 26 *Xiphophorus* species, phylogenetic relationships solely for the northern swordtail clade consisting of nine species (*X. birchmanni*, *X. continens*, *X. cortezi*, *X. malinche*, *X. nezahualcoyotl*, *X. montezumae*, *X. nigrensis* and *X. pygmaeus*) with two outgroups, *X. gordoni* and *X. evelynae* (northern and southern platies that are sister taxa) were reconstructed. This analysis was performed in an effort to resolve the phylogenetic relationships within this group only because the phylogenetic relationships of the northern swordtails were weakly supported and conflicts on the tree topologies were found among different phylogenetic inferences in the whole species tree (Figure 2). The same sequence matrix was used except that columns with gaps only caused by the other species were deleted. Therefore, a total length of 1235 bp (mitochondrial phylogeny, Figure 3a) and 7073 bp (nuclear phylogeny, Figure 3b) sequences was used for these phylogenetic analyses of the northern swordtail group. We also reconstructed a phylogenetic tree using a combined mitochondrial and nuclear data set (8308 bp) (Figure 3c).

The sequence alignment was carried out using the Clustal-W multiple sequences alignment package [85] implemented in BioEdit 7.0 [86] that was then manually adjusted by eye (Additional file 9). We conducted Bayesian Inference (BI), Maximum-Likelihood (ML), Neighbor-Joining (NJ), and Maximum Parsimony (MP) analyses for the phylogenetic reconstruction. These analyses were performed separately for the two combined data sets of two mitochondrial and eleven nuclear genes. The MP and NJ analyses were conducted using MEGA 4.0 [69]. Bootstrap probabilities were obtained with 1000 replicates [87]. For ML and BI analyses, best models of nucleotide substitutions were tested separately for mtDNA and nuclear DNA data sets using jModeltest 0.1.1 [70] under the Akaike Information Criterion (AICc) using a corrected version for small samples [88]. In cases where the models selected by jModeltest were not available in the phylogenetic construction program, the next fit model was applied (Table 1).

ML analyses were performed using PhyML 3.0 [89] and statistical support was obtained with 1000 bootstrap replicates. A Bayesian Markov Chain Monte Carlo approach [90,91] was used as implemented in MrBayes 3.1.2 [92,93]. Three partition schemes including 1) each locus separated, 2) coding and noncoding, and 3) no partition were tested in PartitionFinder 1.0. [94]. The partition for each locus separated was selected as the best scheme under AICc criterion. Two chains were run for 10,000,000 generations, starting from random trees that were sampled every ten generations yielding 10,000,000 trees and the first 25% of trees were discarded as burn-in. An average standard deviation of split frequencies for both runs was less than 0.01, suggesting that concurrent runs converged. Potential scale reduction factor (PSRF) of 1.0 was found, which verifies that we have reliable samples from the posterior probability distribution.

The confidence of phylogenetic tree selection was accessed by the Approximately Unbiased (AU) and Shimodaira-Hasegawa (SH) tests implemented in CONSEL [72]. To do so, the log-likelihoods of site-patterns of the best ML unconstrained trees (Figure 2) were estimated using Tree-Puzzle [95] under the ‘user defined trees’ mode, assuming GTR+G model for nuclear DNA phylogeny and HKY+G+I model for mtDNA phylogeny. Then both the mitochondrial and nuclear best ML constrained trees regarding the positions of *X. monticolus* and *X. clemenciae* were reconstructed using RAxML with constraint option (see the result) [96]. Finally, the best ML unconstrained and constrained trees were compared with each data set in CONSEL [72]. The confidence for each tree topology was presented as *p*-value (Table 2). Additionally, exhaustive search of the ML tree for the northern swordtail group was performed. A strongly supported node (100 of bootstrap value) consisting of three species (*X. multilineatus*, *X. nigrensis* and *X. pygmaeus*) was constrained to reduce the number of OTUs due to computational constraints.

### Ancestral reconstructions of the sword

Ancestral states of swords were reconstructed under parsimony and maximum-likelihood approaches implemented in Mesquite 2.72 [97]. The swords are a composite character including the extension of the caudal fin, coloration of the sword and black pigmentation of the ventral margin [7,20]. Here, we used sword characters as defined in Meyer [26] (characters 1–3), Wiens and Morris [27] (character 4) to include more characters of the sword than only the length of sword. Detailed descriptions of our sword scorings are as follows.

In our maximum-likelihood reconstruction, for character 1 (a two-state) any length of caudal extension was considered as a sword [26]. Characters 2 and 3 were scored as a three-state character (no sword-protrusion-sword) [26]. Character 2 coded *X. andersi* as sworded, but as a protrusion in character 3. Character 4 adopted colored elongation as a sword [27]. Further, we added one more character to consider the polymorphic state of the colored protrusion (character 5). This was applied for a more comprehensive sword scoring based on previous studies [26,27] and our field observation in nature. For character 5 a colored extension was considered as a sword, and species showing a short sword (i.e. protrusion), colored protrusion, or polymorphism for a protrusion or coloration were assigned to the “intermediate” category. However, this scoring is the same as described for character 3 (i.e. intermediate instead of protrusion) so here we do not show the reconstruction result for character 5 separately (see character 3) (Figure 4c).

In our parsimony approach, we used the same scorings as for characters 1–5 as in the maximum likelihood method (Additional file 4). We also added one more character (character 6) in order to apply the polymorphic state of the colored caudal extension since such polymorphic states can only be used in parsimony analyses in Mesquite [97]. Three northern swordtails (*X. birchmanni, X. continens*, and *X. pygmaeus*) and *X. xiphidium* were also coded as polymorphic for the colored caudal extension according to Meyer [26] and our field observations (character 6, Additional file 4). In addition to the ancestral state reconstruction of the sword itself, the ancestral state of other sword traits (coloration: character 7 or ventral black margin: character 8) were also reconstructed separately (Additional file 5) [26]. Coloration of the sword in *X. continens* was also coded as polymorphic because sub-dominant males have an unpigmented protrusion in the wild (character 7; Additional file 5).

The evolutionary history of each of character states was reconstructed based on what would be currently the best phylogenetic hypothesis for this genus obtained by maximum-likelihood inference. The character states were treated as “ordered” and “unordered” in the parsimony reconstructions. The unordered transition state permits the transformation of a character state to any other state. The ML reconstruction was performed with the MK1 (Markov k-state 1 parameter model) model of evolution, which is a k-state generalization of the Jukes-Cantor model and under this model any particular character state changes have equal probability [98,99]. Mesquite does not allow performing ML calculation on the characters that have polymorphism for some taxa. Therefore, only parsimony analyses were performed on the character 6, 7 and 8 (Additional files 4, 5).

## Competing interests

The authors declare that they have no competing interests.

## Authors’ contributions

JK conceived and designed the research, acquired all the molecular data, conducted all the statistical and phylogenetic analyses, and drafted the manuscript. MS contributed samples for the study and commented on the manuscript. RBW provided unpublished DNA sequences and commented on the manuscript. AM conceived and designed the research, participated in the coordination of the study and wrote the manuscript. All authors read and approved the final manuscript.

## Supplementary Material

Additional file 1 **Combined mitochondrial and nuclear phylogeny of the genus *Xiphophorus*. **The phylogenetic tree was constructed from combined sequences (8515 bp) of two mitochondrial and eleven nuclear loci. Numbers above the nodes indicate Bayesian posterior probabilities and Maximum-Likelihood bootstrap values, respectively. Click here for file

Additional file 2 **Maximum-Likelihood trees of the eleven individual nuclear loci (PhyML 3.0).** Detailed information for each locus (i.e., evolutionary substitution models) is shown in Table 1. Maximum-Likelihood bootstrap values higher than 50 are shown. Click here for file

Additional file 3 **Nuclear phylogenies based on two sets of nuclear loci suggest monophyly or paraphyly of the southern swordtails. **The phylogenetic trees were constructed from (a) combined sequences of seven nuclear loci including D8, X-*src*, Rag1, GNG13, G6PD, POLB and FEN1 (5166 bp) and (b) combined sequences of four nuclear loci including UNG, TP53, T36 and D2 (2110 bp). The full name of these nuclear loci is given in Table 1. Numbers above the nodes indicate Bayesian posterior probabilities, Maximum-Likelihood, Neighbor-Joining and Maximum-Parsimony bootstrap values, respectively. The total length of aligned sequences for the first seven loci combined was 5166 bp with 0.015 (SE = 0.001) average nucleotide diversity (*p*-distance) and 267 sites were parsimony informative among 449 variable sites. The second four loci combined included 2110 bp with 0.023 (SE = 0.002) and 145 sites were parsimony informative among 241 variable sites. TVM+G and GTR+G were chosen as the best evolutionary models for the first and second classes of genes, respectively. Click here for file

Additional file 4 **Parsimony reconstructions for the ancestral state of the sword in the genus *Xiphophorus*. **Six different characters were mapped onto a nuclear tree: (a) a two-state character of any length of caudal extension (character 1), (b) a three-state character of sword extension (no sword, protrusion and sword; *X. andersi *was coded as a sworded species, character 2), (c) a three-state character of sword extension (*X. andersi *was coded as a species with protrusion, character 3), (d) a two-state character of colored caudal extension (character 4), (e) a three-state character of colored caudal extension with intermediate state (character 5), and (f) colored caudal extension with polymorphic state (character 6). Each circle on the nodes represents character state (black filled circles: sword; green circles: protrusion (characters 2 and 3) or intermediate within species (characters 5); empty circles: no sword; grey circles: unknown). Transition state was treated as “ordered” (i.e., protrusion is an intermediated step from ‘no sword’ to ‘sword’ states) or “unordered”. Click here for file

Additional file 5 **Parsimony reconstructions for the ancestral state of (a) coloration (character 7) and (b) ventral black margin (character 8) in caudal fin in the genus*Xiphophorus*. **Each circle on the node represents the character state (black filled circles: presence of the character; half black filled circles: polymorphic; empty circles: absence of the character). Transition state was treated as “ordered” (i.e., polymorphic is an intermediated step from ‘absent’ to ‘present’ states) or “unordered”. Click here for file

Additional file 6 **Ancestral states of the sword in the genus *Xiphophorus *by maximum-likelihood method. **This table presents the proportional likelihoods for each node of phylogeny. Click here for file

Additional file 7 **Specimen information. **This table shows a list of specimens, sample localities, accession numbers of nuclear and mitochondrial DNA sequences used in this study. Click here for file

Additional file 8 **Primer information. **This table provides DNA sequences and PCR conditions of newly developed primers. Click here for file

Additional file 9 DNA sequence alignment of two mitochondrial and eleven nuclear loci. Click here for file
