# Supplementary figures and images for: Comprehensive phylogenetic analysis of all species of swordtails and platies (Pisces: Genus Xiphophorus) uncovers a hybrid origin of a swordtail fish, Xiphophorus monticolus, and demonstrates that the sexually selected sword originated in the ancestral lineage of the genus, but was lost again secondarily
Source: BMC Evol Biol. 2013 Jan 29;13:25. doi: 10.1186/1471-2148-13-25 (PMC3585855; doi:10.1186/1471-2148-13-25)

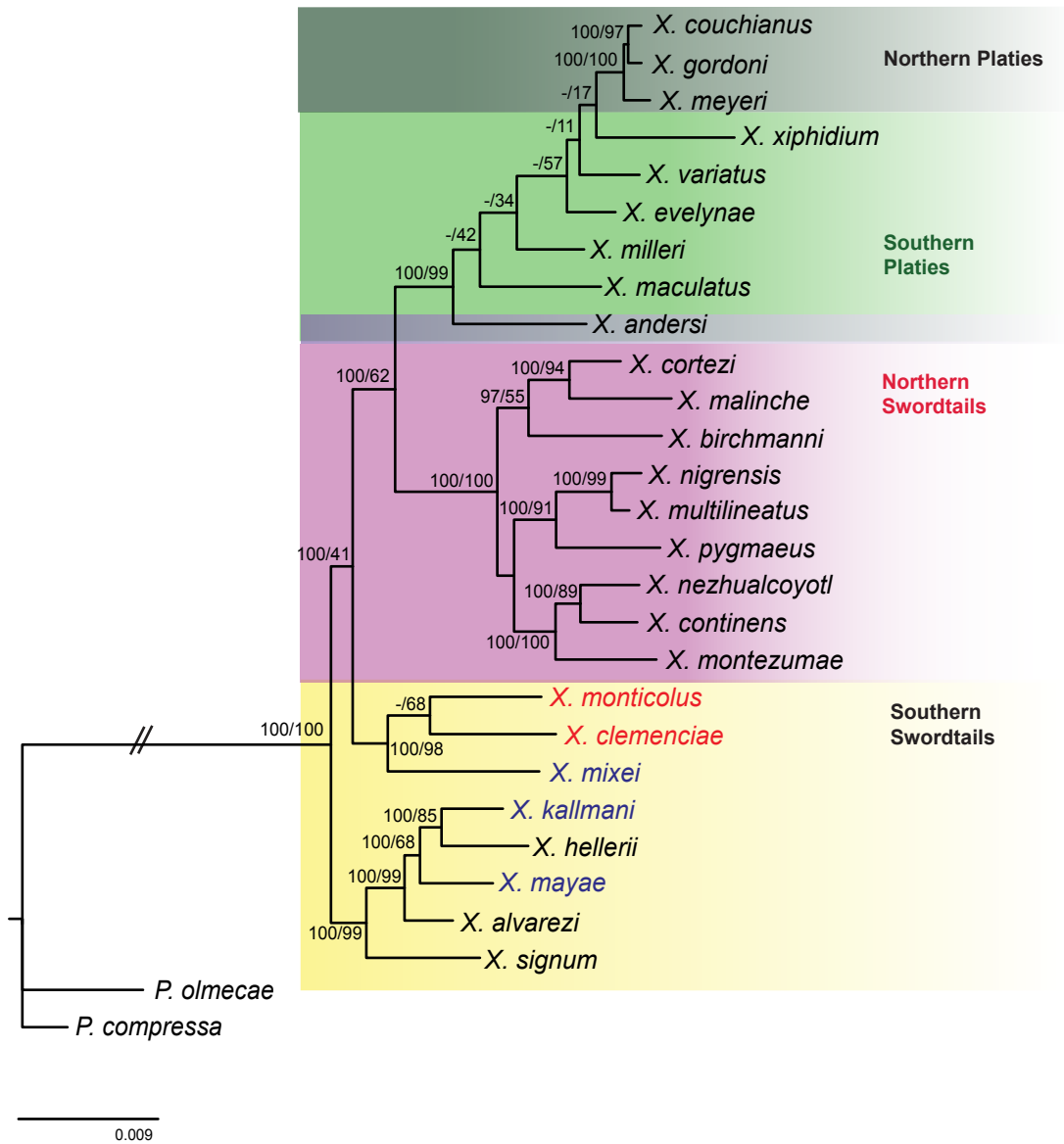

Supplement: Additional file 1 — Combined mitochondrial and nuclear phylogeny of the genus Xiphophorus. The phylogenetic tree was constructed from combined sequences (8515 bp) of two mitochondrial and eleven nuclear loci. Numbers above the nodes indicate Bayesian posterior probabilities and Maximum-Likelihood bootstrap values, respectively. [file 1471-2148-13-25-S1.pdf]

D2

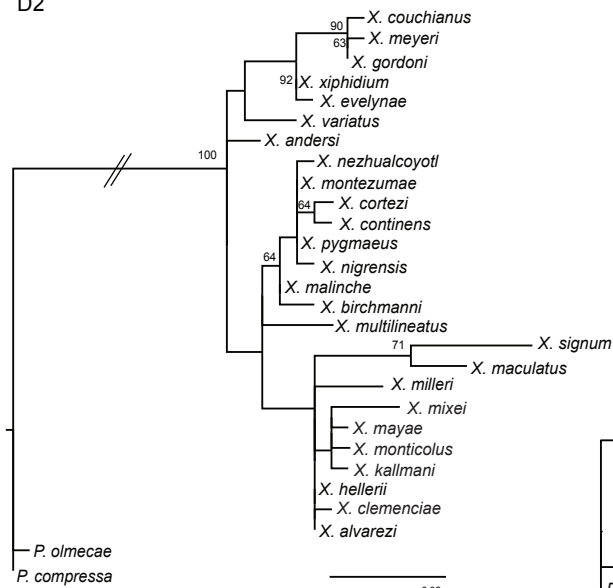

D8

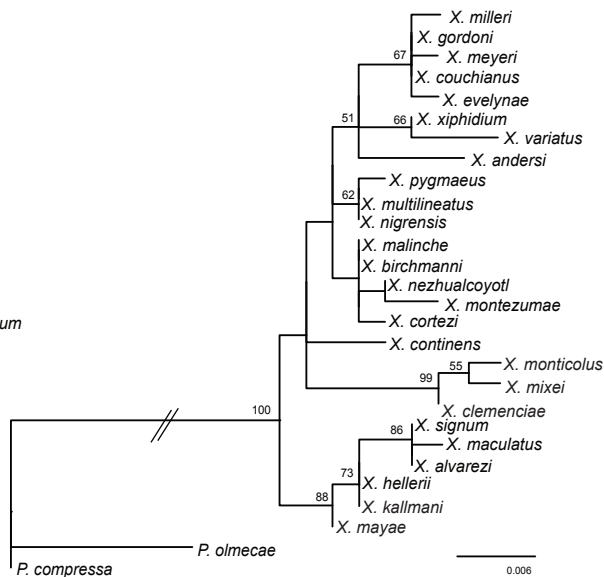

T36

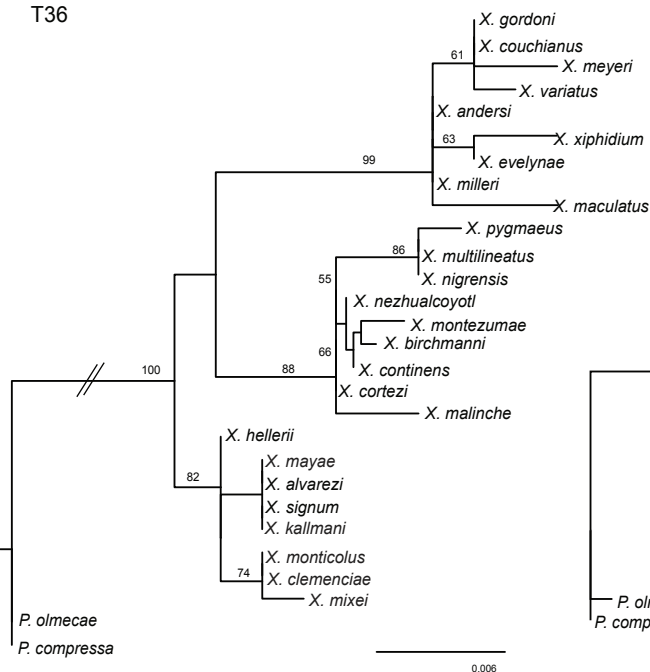

Xsrc

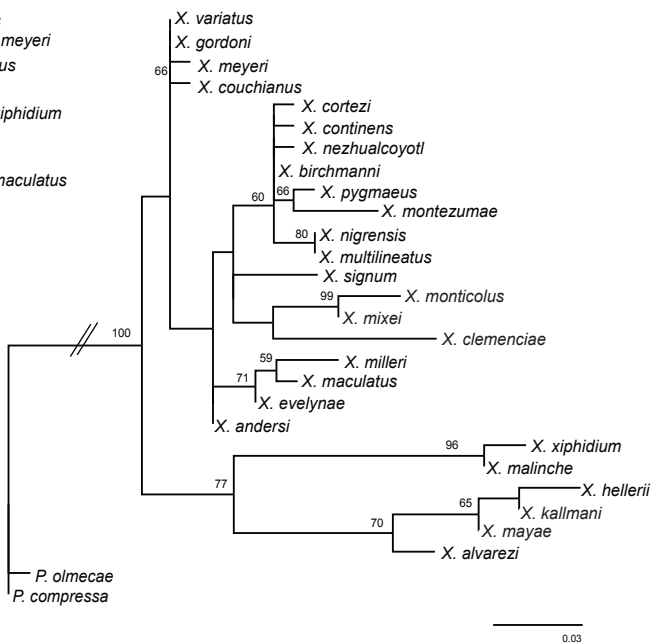

Rag1

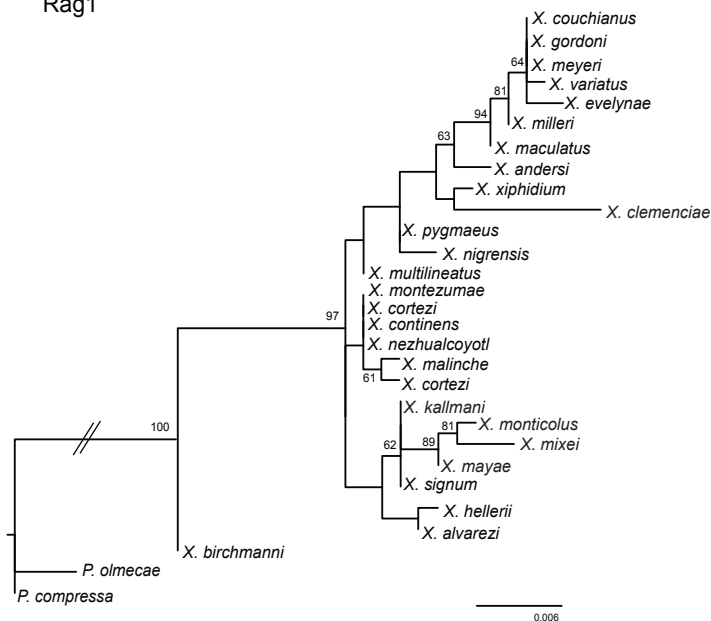

GNG13C2

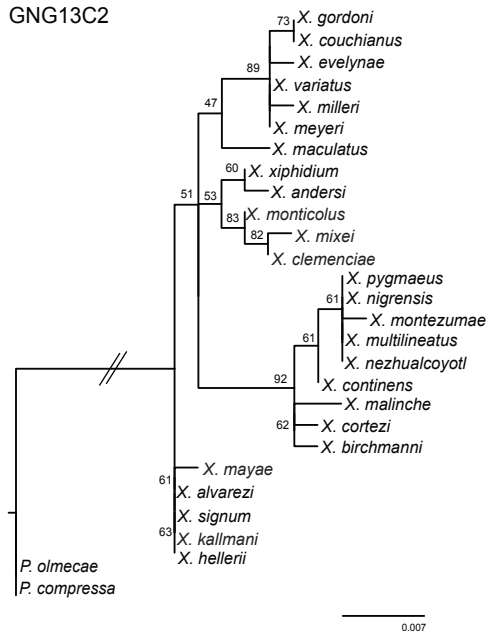

G6PD

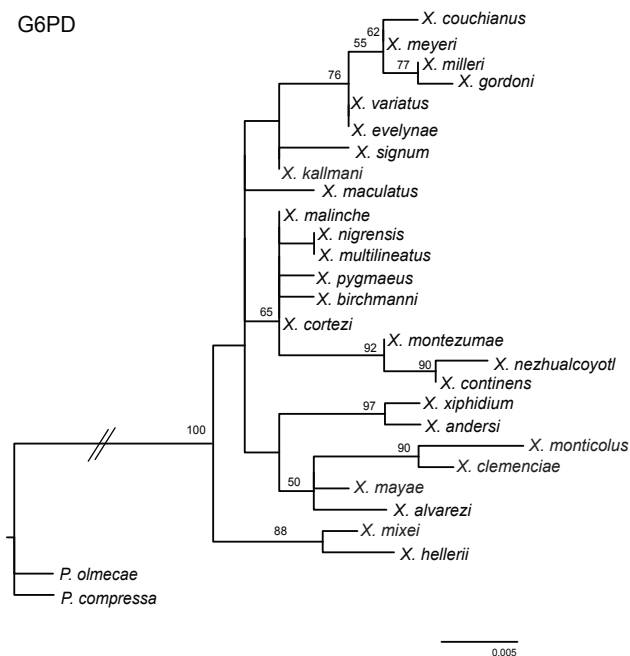

UNG

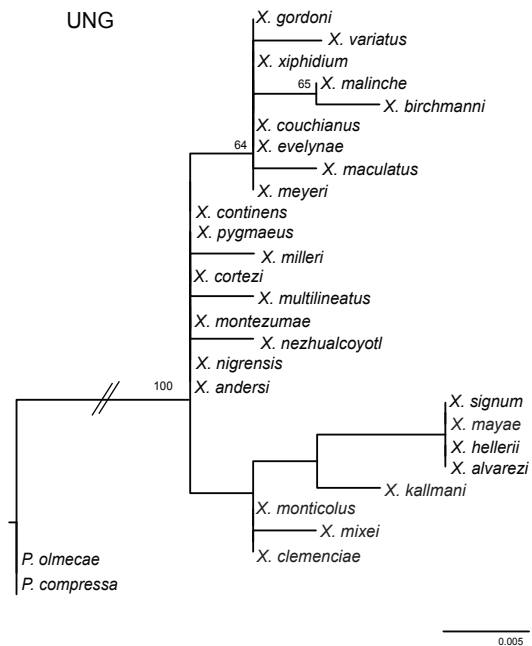

POLB

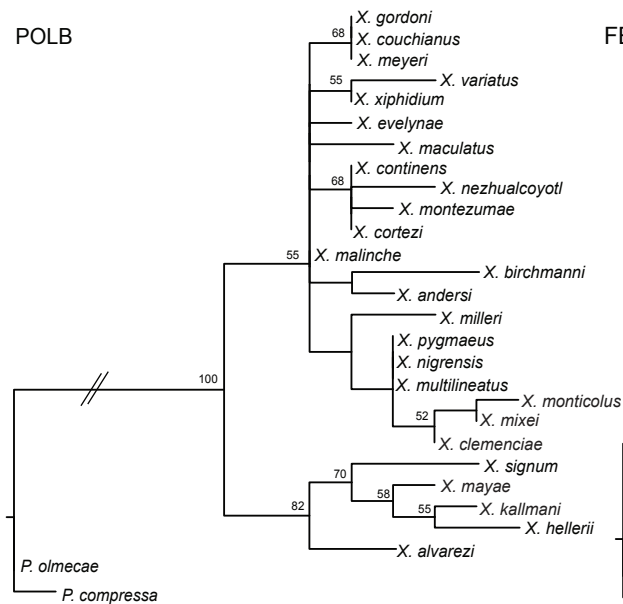

FEN1

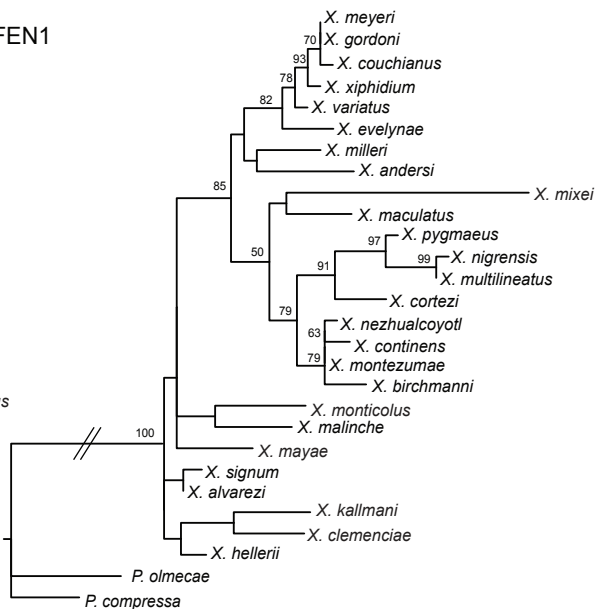

TP53

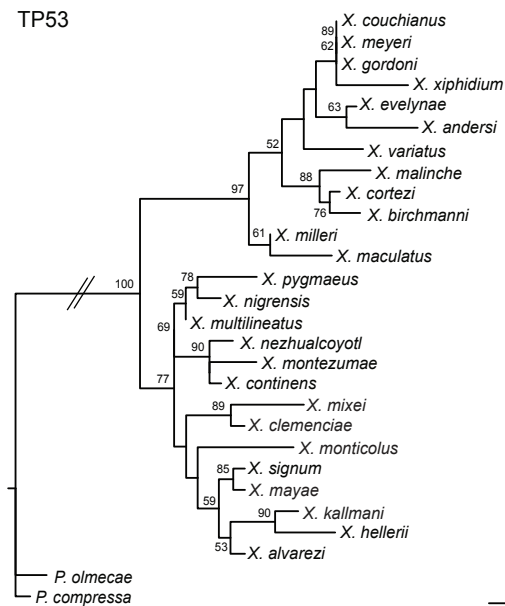

Supplement: Additional file 2 — Maximum-Likelihood trees of the eleven individual nuclear loci (PhyML 3.0). Detailed information for each locus (i.e., evolutionary substitution models) is shown in Table 1. Maximum-Likelihood bootstrap values higher than 50 are shown. [file 1471-2148-13-25-S2.pdf]

(a) paraphyly of the southern swordtails

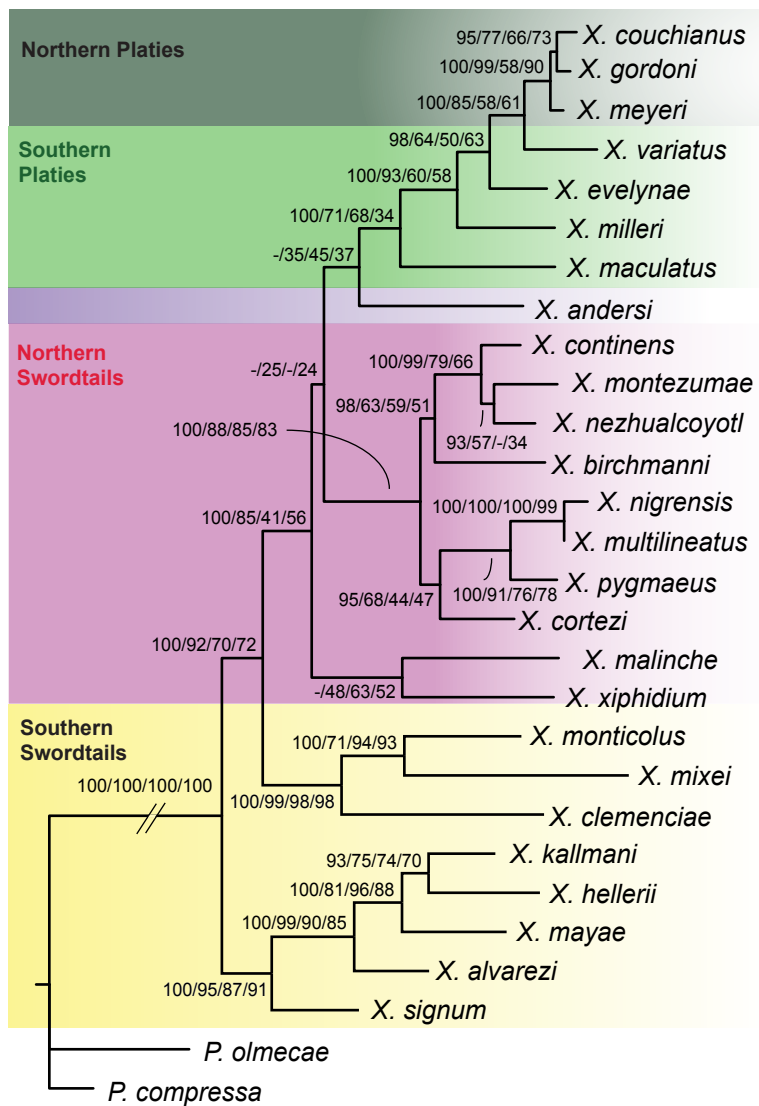

(b) monophyly of the southern swordtails

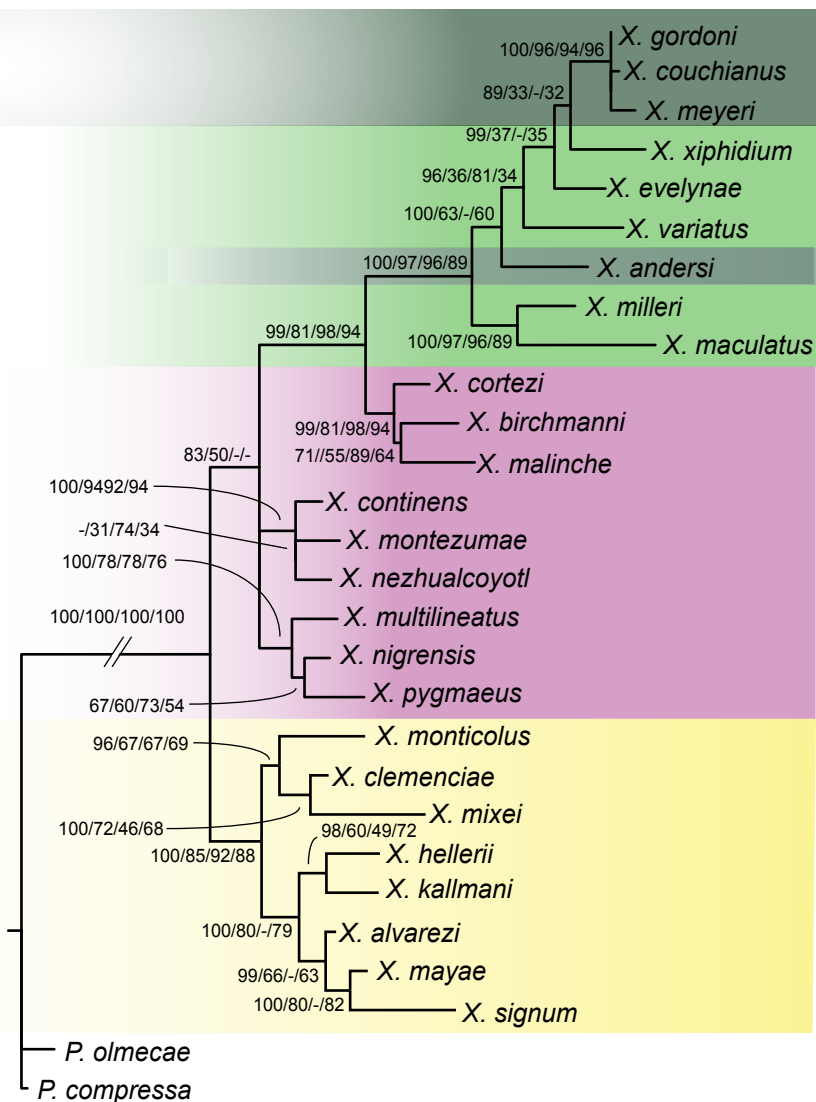

Supplement: Additional file 3 — Nuclear phylogenies based on two sets of nuclear loci suggest monophyly or paraphyly of the southern swordtails. The phylogenetic trees were constructed from (a) combined sequences of seven nuclear loci including D8, X-src, Rag1, GNG13, G6PD, POLB and FEN1 (5166 bp) and (b) combined sequences of four nuclear loci including UNG, TP53, T36 and D2 (2110 bp). The full name of these nuclear loci is given in Table 1. Numbers above the nodes indicate Bayesian posterior probabilities, Maximum-Likelihood, Neighbor-Joining and Maximum-Parsimony bootstrap values, respectively. The total length of aligned sequences for the first seven loci combined was 5166 bp with 0.015 (SE = 0.001) average nucleotide diversity (p-distance) and 267 sites were parsimony informative among 449 variable sites. The second four loci combined included 2110 bp with 0.023 (SE = 0.002) and 145 sites were parsimony informative among 241 variable sites. TVM+G and GTR+G were chosen as the best evolutionary models for the first and second classes of genes, respectively. [file 1471-2148-13-25-S3.pdf]
