# Supplementary material for: Comprehensive phylogenetic analysis of all species of swordtails and platies (Pisces: Genus Xiphophorus) uncovers a hybrid origin of a swordtail fish, Xiphophorus monticolus, and demonstrates that the sexually selected sword originated in the ancestral lineage of the genus, but was lost again secondarily
Source: BMC Evol Biol. 2013 Jan 29;13:25. doi: 10.1186/1471-2148-13-25 (PMC3585855; doi:10.1186/1471-2148-13-25)

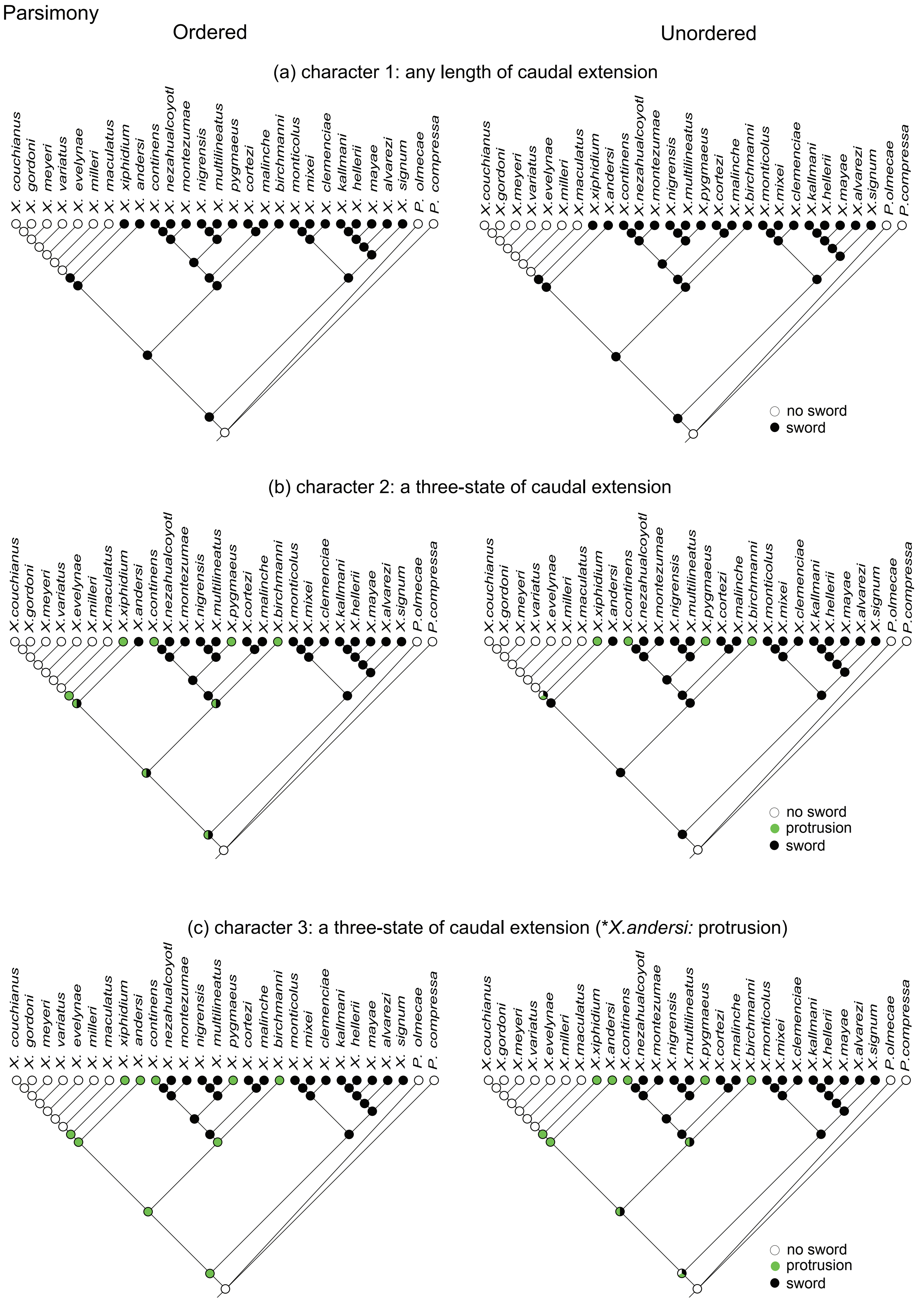

Parsimony
Ordered
Unordered
(a) character 1: any length of caudal extension
X. couchianus X. gordoni X. meyeri X. variatus X. evelynae X. milleri X. maculatus X. xiphidium X. andersi X. continens X. nezahualcoyotl X. montezumae X. nigrensis X. multilineatus X. pygmaeus X. cortezi X. malinche X. birchmanni X. monticolus X. mixei X. clemenciae X. kallmani X. hellerii X. mayae X. alvarezi X. signum P. olmecae P. compressa
X.couchianus X.gordoni X.meyeri X.variatus X.evelynae X.milleri X.maculatus X.xiphidium X.andersi X.continens X.nezahualcoyotl X.montezumae X.nigrensis X.multilineatus X.pygmaeus X.cortezi X.malinche X.birchmanni X.monticolus X.mixei X.clemenciae X.kallmani X.hellerii X.mayae X.alvarezi X.signum P.olmecae P.compressa
no sword
sword
(b) character 2: a three-state of caudal extension
X.couchianus X.gordoni X.meyeri X.variatus X.evelynae X.milleri X.maculatus X.xiphidium X.andersi X.continens X.nezahualcoyotl X.montezumae X.nigrensis X.multilineatus X.pygmaeus X.cortezi X.malinche X.birchmanni X.monticolus X.mixei X.clemenciae X.kallmani X.hellerii X.mayae X.alvarezi X.signum P.olmecae P.compressa
X.couchianus X.gordoni X.meyeri X.variatus X.evelynae X.milleri X.maculatus X.xiphidium X.andersi X.continens X.nezahualcoyotl X.montezumae X.nigrensis X.multilineatus X.pygmaeus X.cortezi X.malinche X.birchmanni X.monticolus X.mixei X.clemenciae X.kallmani X.hellerii X.mayae X.alvarezi X.signum P.olmecae P.compressa
no sword
protrusion
sword
(c) character 3: a three-state of caudal extension (*X.andersi: protrusion)
X. couchianus X. gordoni X. meyeri X. variatus X. evelynae X. milleri X. maculatus X. xiphidium X. andersi X. continens X. nezahualcoyotl X. montezumae X. nigrensis X. multilineatus X. pygmaeus X. cortezi X. malinche X. birchmanni X. monticolus X. mixei X. clemenciae X. kallmani X. hellerii X. mayae X. alvarezi X. signum P. olmecae P. compressa
X.couchianus X.gordoni X.meyeri X.variatus X.evelynae X.milleri X.maculatus X.xiphidium X.andersi X.continens X.nezahualcoyotl X.montezumae X.nigrensis X.multilineatus X.pygmaeus X.cortezi X.malinche X.birchmanni X.monticolus X.mixei X.clemenciae X.kallmani X.hellerii X.mayae X.alvarezi X.signum P.olmecae P.compressa
no sword
protrusion
sword

Parsimony

Ordered | Unordered

(d) character 4: colored caudal extension

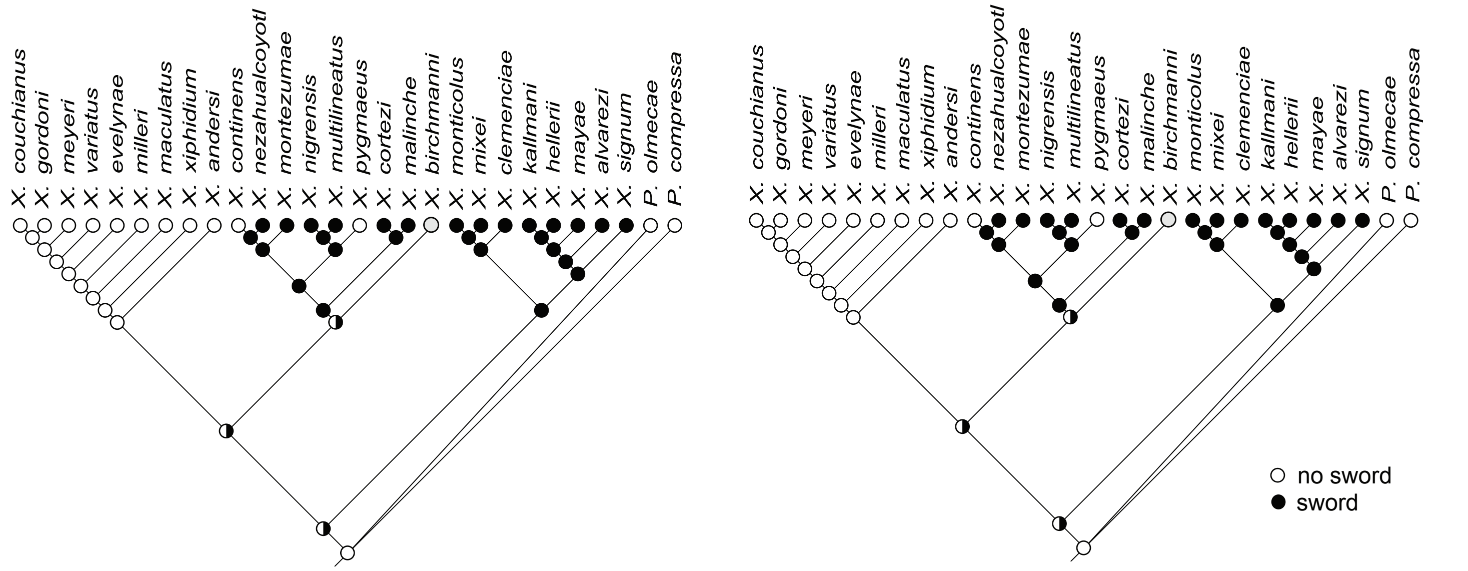

(e) character 5: colored caudal extension with intermediate state

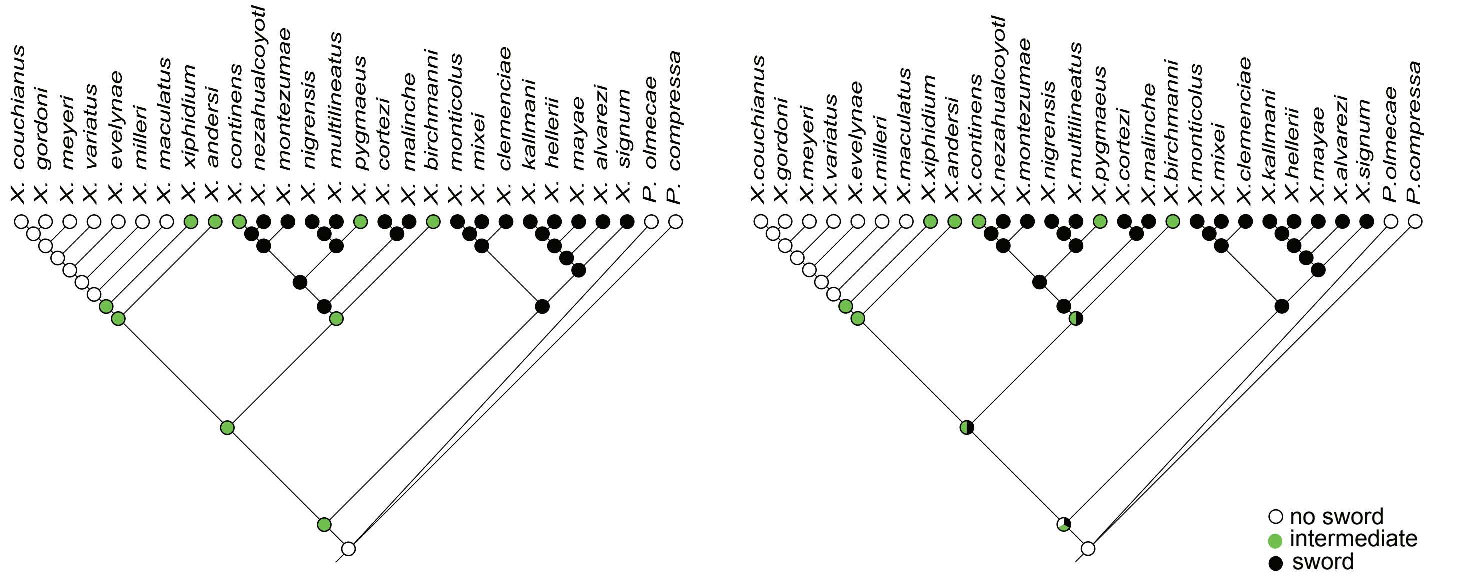

(f) character 6: colored caudal extension with polymorphic state

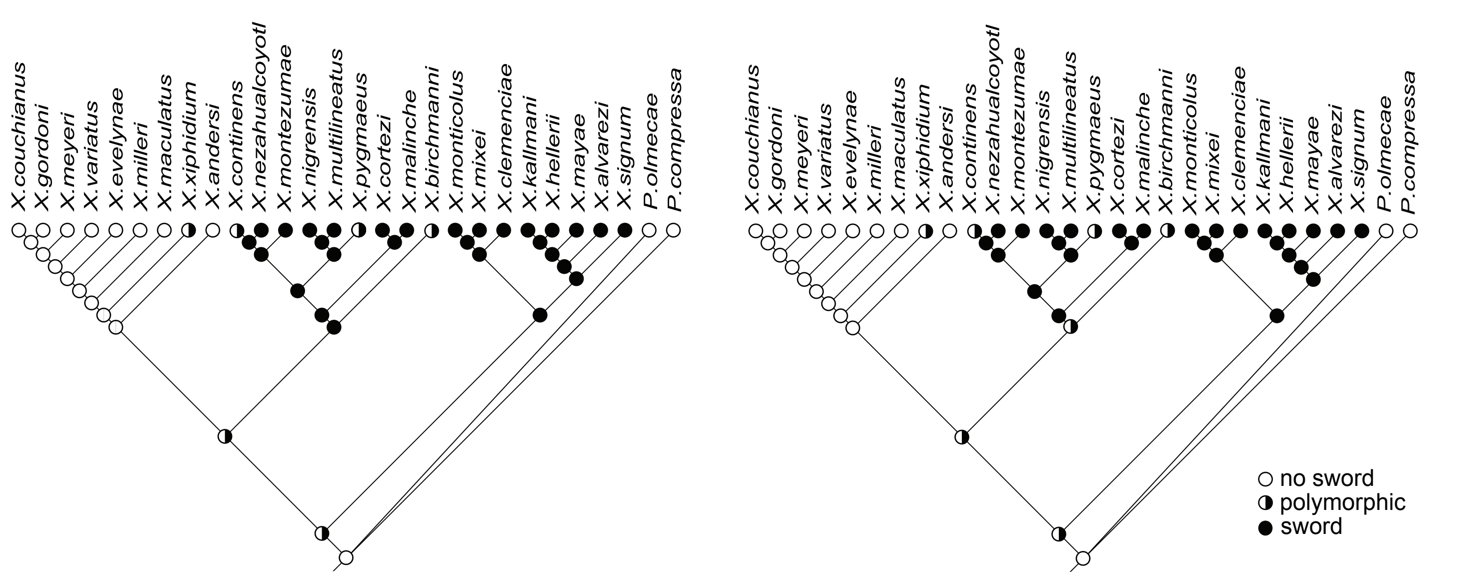

Supplement: Additional file 4 — Parsimony reconstructions for the ancestral state of the sword in the genus Xiphophorus. Six different characters were mapped onto a nuclear tree: (a) a two-state character of any length of caudal extension (character 1), (b) a three-state character of sword extension (no sword, protrusion and sword; X. andersi was coded as a sworded species, character 2), (c) a three-state character of sword extension (X. andersi was coded as a species with protrusion, character 3), (d) a two-state character of colored caudal extension (character 4), (e) a three-state character of colored caudal extension with intermediate state (character 5), and (f) colored caudal extension with polymorphic state (character 6). Each circle on the nodes represents character state (black filled circles: sword; green circles: protrusion (characters 2 and 3) or intermediate within species (characters 5); empty circles: no sword; grey circles: unknown). Transition state was treated as “ordered” (i.e., protrusion is an intermediated step from ‘no sword’ to ‘sword’ states) or “unordered”. [file 1471-2148-13-25-S4.pdf]
