# Supplementary material for: Comprehensive phylogenetic analysis of all species of swordtails and platies (Pisces: Genus Xiphophorus) uncovers a hybrid origin of a swordtail fish, Xiphophorus monticolus, and demonstrates that the sexually selected sword originated in the ancestral lineage of the genus, but was lost again secondarily
Source: BMC Evol Biol. 2013 Jan 29;13:25. doi: 10.1186/1471-2148-13-25 (PMC3585855; doi:10.1186/1471-2148-13-25)

Parsimony

Ordered | Unordered

(a) character 7: coloration of caudal fin

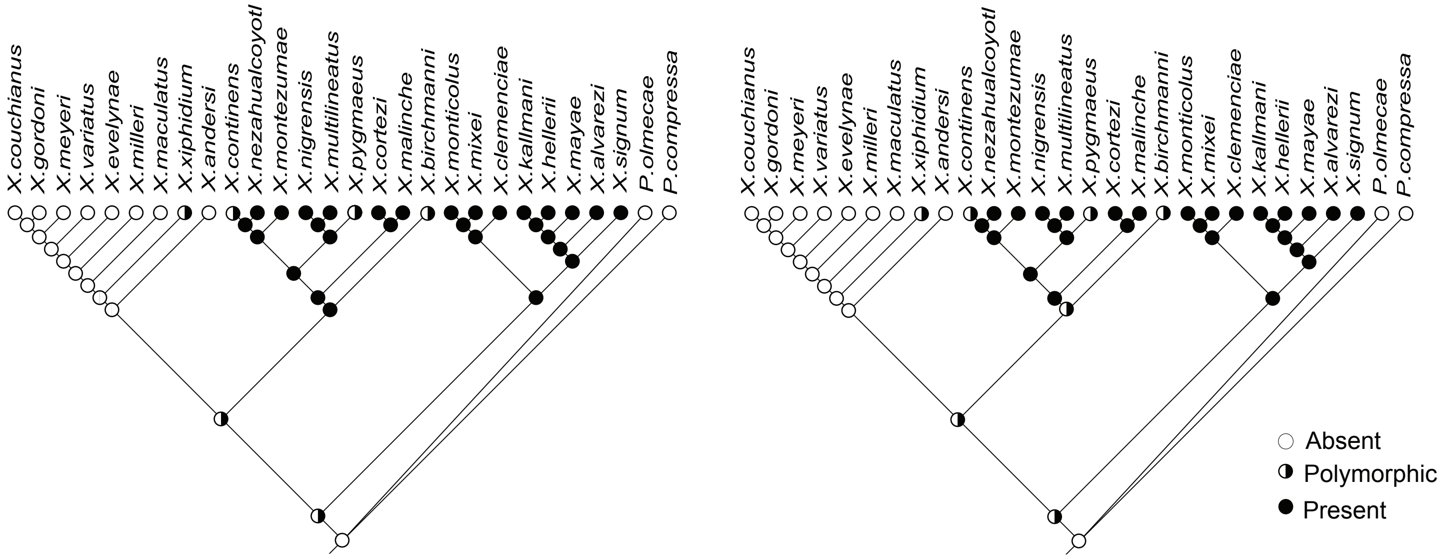

(b) character 8: ventral black margin of caudal fin

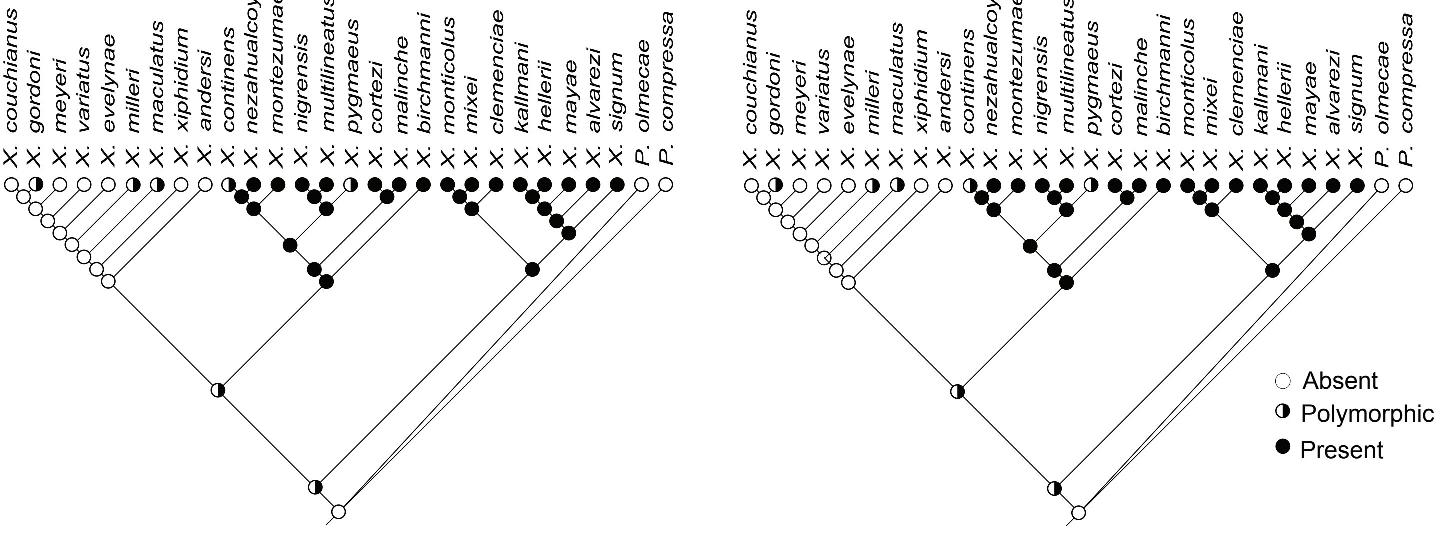

Supplement: Additional file 5 — Parsimony reconstructions for the ancestral state of (a) coloration (character 7) and (b) ventral black margin (character 8) in caudal fin in the genusXiphophorus. Each circle on the node represents the character state (black filled circles: presence of the character; half black filled circles: polymorphic; empty circles: absence of the character). Transition state was treated as “ordered” (i.e., polymorphic is an intermediated step from ‘absent’ to ‘present’ states) or “unordered”. [file 1471-2148-13-25-S5.pdf]
