# Supplementary material for: Comprehensive phylogenetic analysis of all species of swordtails and platies (Pisces: Genus Xiphophorus) uncovers a hybrid origin of a swordtail fish, Xiphophorus monticolus, and demonstrates that the sexually selected sword originated in the ancestral lineage of the genus, but was lost again secondarily
Source: BMC Evol Biol. 2013 Jan 29;13:25. doi: 10.1186/1471-2148-13-25 (PMC3585855; doi:10.1186/1471-2148-13-25)
Supplement: Additional file 6 — Ancestral states of the sword in the genus Xiphophorus by maximum-likelihood method. This table presents the proportional likelihoods for each node of phylogeny. [file 1471-2148-13-25-S6.doc]

**Additional file 6 Ancestral states of the sword in the genus *Xiphophorus* by maximum-likelihood method.**

| State | Node | 1(a) | 2(b) | 3, 5 (c) | 4 (d) |
| --- | --- | --- | --- | --- | --- |
|  | -log L | 8.284 | 21.108 | 21.456 | 13.437 |
| No sword | All *Xiphophorus* | 0.004 | 0.024 | 0.040 | 0.032 |
| Protrusion |  | - | 0.011 | 0.039 | - |
| Sword |  | **0.996** | **0.965** | **0.921** | **0.967** |
| No sword | Platies | 0.039 | 0.004 | 0.448 | **0.949** |
| Protrusion |  | **-** | 0.002 | 0.382 | - |
| Sword |  | **0.961** | **0.993** | 0.170 | 0.050 |
| No sword | Northern swordtails | 0.000 | 0.025 | 0.041 | - |
| Protrusion |  | - | 0.049 | 0.146 | - |
| Sword |  | **0.999** | **0.926** | 0.813 | - |
| No sword | Platies + Northern swordtails | 0.007 | 0.010 | 0.167 | 0.124 |
| Protrusion |  | - | 0.032 | 0.197 | - |
| Sword |  | **0.993** | **0.867** | 0.637 | 0.875 |
| No sword | Southern swordtails | 0.000 | 0.004 | 0.008 | 0.007 |
| Protrusion |  | **-** | 0.002 | 0.008 | - |
| Sword |  | **0.999** | **0.993** | **0.983** | **0.992** |

Ancestral state at each node is shown with five different characters: (a) a two-state character of sword extension (character 1), (b) a three-state character of sword extension (no sword, protrusion and sword; *X. andersi* was coded as a sworded species, character 2), (c) a three-state character of sword extension (*X. andersi* was coded as a species with protrusion, character 3) and colored caudal extension with intermediate state (character 5), and (d) a two-state colored caudal extension. The proportional likelihoods for each node are shown and the states judged best according to the threshold as shown in bold.
