# Supplementary material for: Comprehensive phylogenetic analysis of all species of swordtails and platies (Pisces: Genus Xiphophorus) uncovers a hybrid origin of a swordtail fish, Xiphophorus monticolus, and demonstrates that the sexually selected sword originated in the ancestral lineage of the genus, but was lost again secondarily
Source: BMC Evol Biol. 2013 Jan 29;13:25. doi: 10.1186/1471-2148-13-25 (PMC3585855; doi:10.1186/1471-2148-13-25)
Supplement: Additional file 8 — Primer information. This table provides DNA sequences and PCR conditions of newly developed primers. [file 1471-2148-13-25-S8.doc]

**Additional file 8** DNA sequences of newly designed primer-pairs in this study.

| **Name** | **Locus** | **Forward** | | **Reverse** | | **Tm (**°C**)** |
| --- | --- | --- | --- | --- | --- | --- |
| GNG | Guanine nucleotide binding protein (G protein) gamma 13 (1^st^ intron) | 5'-AAGCCTTCAGTATCAGCTG-3' | 5'-CGCCTTCTCCACCCAGGGGTT-3' | | 45.4 | |
| G6PD | Glucose-6-phosphate dehydrogenase (6^th^ intron) | 5'-CTGGGCAAAGAAATGGTGCA-3' | 5'-AGTATCCTCCTCGCCCCTGA-3' | | 58 | |
| UNG | Uracil-DNA-glycosylase (4^th^ intron) | 5'-TCACAGCGTCAGTGAAGGTC-3' | 5'-AAAGAGCTGGCCACAGACAT-3' | | 58 | |
| POLB | DNA polymerase beta (7^th^ to 11^th^ intron) | 5'-CCACCAAAAGATTGGACTCAA-3' | 5'-TGGTGTCTCCTTTGGACAGA-3' | | 52 | |
| FEN1 | Flap structure-specific endonuclease 1 (3^rd^ intron) | 5'-ACCACCTCTGGCTTCAAAAA-3' | 5'-TGTATTCTGCTGGGGTGTGA-3' | | 52 | |
| TP53 | Tumor protein p53 (4^th^ intron) | 5'-GATCCGGAAACTATGCAGGA-3' | 5'-CCAATTGGAGTCGTTTTTGC-3' | | 52 | |
